# Opposing Effect of Naringenin and Quercetin on the Junctional Compartment of MDCK II Cells to Modulate the Tight Junction

**DOI:** 10.3390/nu12113285

**Published:** 2020-10-27

**Authors:** Mio Nakashima, Misaki Hisada, Natsuko Goda, Takeshi Tenno, Ayaka Kotake, Yuko Inotsume, Ikuo Kameoka, Hidekazu Hiroaki

**Affiliations:** 1 Graduate School of Pharmaceutical Sciences, Nagoya University, Furocho, Chikusa, Nagoya, Aichi 464-8601, Japan; nakashima.mio@a.mbox.nagoya-u.ac.jp (M.N.); hisada.misaki@gmail.com (M.H.); tenno.natsuko@f.mbox.nagoya-u.ac.jp (N.G.); tenno.takeshi@e.mbox.nagoya-u.ac.jp (T.T.); 2 Department of Biological Sciences, Faculty of Science, Nagoya University, Furocho, Chikusa, Nagoya, Aichi 464-8602, Japan; 3 BeCerllBar, LLC., Business Incubation Building, Nagoya University, Furocho, Chikusa-ku, Nagoya, Aichi 464-8601, Japan; 4 Cosmetics Research Department, Nicca Chemical Co. Ltd., Fukui 910-8670, Japan; a-kotake@demicosmetics.com (A.K.); y-inotsume@demicosmetics.com (Y.I.); i-kameoka@niccachemical.com (I.K.)

**Keywords:** dynamic equilibrium of tight junction, flavonoids, epithelial barrier enhancer, 5′ adenosine monophosphate (AMP)-activated protein kinase, phosphatidylinositol-3-phosphate kinase, tight junction integrity

## Abstract

Maintaining tight junction (TJ) integrity is important for epithelial cell barriers. Previously, the enhancement of TJ integrity, induced by citrus-derived flavonoids, naringin (NRG) and hesperidin (HSD), was demonstrated, but the effects of their aglycones naringenin (NAR) and hesperetin (HST), and the mechanisms, have not been systematically investigated. Here we compared three series of flavonoids related to NAR, HST, quercetin (QUE) and their glycosides with the Madin–Darby canine kidney (MDCK) II cell monolayers. The effect of flavonoids on the protein expression level of claudin (CLD)-2 and its subcellular localization were investigated. NAR, NRG, and HSD increased the CLD-2 localization at the TJ compartment, and its protein expression level. QUE and HST showed TJ-mitigating activity. Narirutin (NRT), neohesperidin (NHD) and rutin (RUT) did not affect the TJ. In addition, NAR and QUE induced an increase or decrease of the transepithelial electrical resistance (TEER) values of the MDCK II monolayers. Two known signaling pathways, phosphatidyl-inositol-3 kinase (PI3K) and 5′-AMP-activated protein kinase (AMPK), were further compared with NAR. Two-dimensional polyacrylamide electrophoresis (2D PAGE) analysis of whole-cell proteins treated with NAR, AICA-riboside (AMPK activator) and LY294002 (PI3K inhibitor) showed in both a distinct pattern. This suggests the target of NAR’s CLD-2 or zonula occludens-1 (ZO-1) modulation was unique.

## 1. Introduction

The tight junction (TJ) is the proteinous complex located at the apical-most region of epithelial and endothelial cells [1,2,3]. The TJ strands are composed of four-transmembrane protein claudins (CLDs) and occludin (OCLN), with cytosolic scaffold zonula occludens (ZO)-1/2/3 proteins connected to cytoskeletal actin [3,4]. The human genome encodes more than 20 CLDs, and the different combinations of CLDs were found to be at the root of the TJ’s unique characteristics [5]. A mature tight junction serves as a barrier to limit the permeation of ions and molecules through paracellular pathways. It also helps maintain cell polarity by limiting the diffusion of membrane proteins and lipid molecules as a “fence” [6]. The TJs develop particularly well in the digestive organs, vascular system, and epidermal cells of the skin, and exert their barrier function. The barrier function varies depending on the physiological role of each organ and type of cell. In addition, inflammation is known to weaken intestinal TJ integrity, with increased permeability of xenobiotics and pathogens through cell monolayers. Disruption of the TJ barrier’s function may allow the pathogens to enter the interior, thereby worsening further inflammation responses. Thus, a strengthening of the TJs by way of nutrients or prophylactic drugs is a beneficial food science application [7,8].

The homeostasis of the TJ is maintained by two opposed cellular processes: promoting biogenesis of the TJ, and internalization followed by degradation of the TJ. For the former process, ZO proteins promote the accumulation of CLDs in the membrane [6,9,10]. ZO proteins are membrane-associated multi-PSD-95, Dlg1, ZO-1 (PDZ) (postsynaptic density 95 (PSD-95), large discs, zonula occludens 1) domain-containing proteins. As the latter process, conversely, ligand of Numb protein X1 p80 (LNX1p80), one of the splicing variants of the PDZ-domain, containing E3 ubiquitin ligase LNX1 (Ligand of Numb X1), promotes CLD internalization and lysosomal degradation by ubiquitination, depending on their manner, thereby causing TJ downregulation [11]. Both ZO proteins and LNX1 bind to the C-terminus of CLDs, likely via their PDZ domains, suggesting that their competitive interactions with CLDs could control TJ integrity.

**Figure 1:**
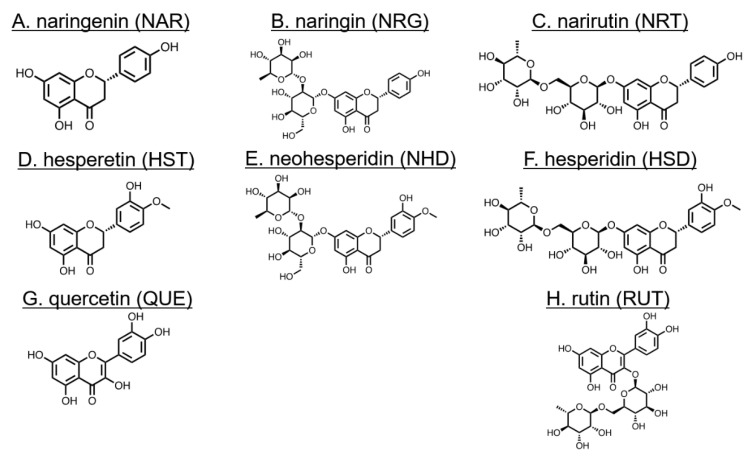
Chemical structures of flavonoids and their abbreviations. (**A**) naringenin (NAR), (**B**) naringin (NRG), (**C**) narirutin (NRT), (**D**) hesperetin (HST), (**E**) neohesperidin (NHD), (**F**) hesperidin (HSD), (**G**) quercetin (QUE), and (**H**) rutin (RUT).

In our previous study, we reported that two flavonoids baicalin and baicalein exhibited TJ-mitigating activity in two model epithelial cells, Madin–Darby canine kidney (MDCK) II cell and Caco-2 cell [12]. We demonstrated the direct interaction between these flavonoids and the first PDZ domain of ZO-1 (ZO-1(PDZ1)). When we examined 12 flavonoids, including baicalin and baicalein, we preliminarily found that naringenin (NAR) (Figure 1) exhibited an enhanced subcellular localization of CLD-2 in the intercellular junctional compartment in MDCK II cells. Although an improvement in TJ integrity or intestinal barrier function by the related citrus-derived flavonoids naringenin (NAR), naringin (NRG) and hesperetin (HST) was reported [13,14,15,16,17], systematic comparative studies of the series of flavonoids of common aglycones with diverged glycosides remain a necessary future approach.

In this study, we examined eight flavonoids, including NAR, HST, QUE and their glycosides (Figure 1), focusing on TJ-modulating activity in the epithelial MDCK II cell model. MDCK II, which is often used in TJ research, forms stable and continuous TJs within 24 to 48 h after the cells reaching confluency. Among these flavonoids, NAR showed the strongest TJ-modulating activity with increasing transepithelial electrical resistance (TEER). We also confirmed an increased accumulation of CLD-1, CLD-3 and CLD-7 by NAR. Notably, QUE induced a decrease in CLD-2 expression. QUE treatment also exhibited a change of morphology into a cobblestone-like MDCK II cell, with its slender form resembling a fibroblast. The cellular mechanism, in the action of NAR-induced TJ enhancement, was further studied by a proteomic approach. The result suggested the existence of a yet-unknown signaling pathway.

## 2. Materials and Methods

### 2.1. Materials

HST, hesperidin (HSD), rutin (RUT), QUE, AICAR (5-aminoimidazole-4-carboxamide-1-β-d-ribofuranoside) and LY294002 were purchased from FUJIFILM Wako Pure Chemical Corporation (Osaka, Japan). NAR and NRG were purchased from Tokyo Chemical Industry Co. Ltd. (Tokyo, Japan). NRT and neohesperidin (NHD) were purchased from Cayman Chemical Company (Ann Arbor, MI, USA). All compounds used for NMR and cell experiments were dissolved into d_6_-dimethylsulfoxide (DMSO) as a 10 mM solution and stored at −20 °C until needed.

The rabbit anti-CLD-2 antibody was obtained from Sigma-Aldrich (St. Louis, MO, USA). The rabbit anti-ZO-1 antibody was obtained from Invitrogen (Carlsbad, CA, USA). Mouse anti-β-actin antibody was purchased from Wako. For immunofluorescent microscopy, the anti-rabbit immunoglobulin G (IgG) and the F (ab’) 2 fragment-Cy3 antibody were obtained from Sigma-Aldrich. For Western blotting analysis, anti-rabbit and mouse IgG horseradish peroxidase (HRP) conjugates were acquired from Promega (Madison, WI, USA).

### 2.2. Protein Expression and Purificaion

The expression and purification of the mouse ZO-1(PDZ1) (residues 18–110) was previously described [18]. In brief, the recombinant glutathione-S-transferase (GST)-tagged form of ^15^N-labeled mouse ZO-1(PDZ1) was expressed by *Escherichia coli* BL21 (*DE3*) in 1 L M9 minimal media with ^15^N-ammonium chloride as the sole nitrogen source, supplemented with divalent cations and vitamins at 20 °C. The fusion protein was captured by affinity purification on GST-accept (Nacalai Tesque, Kyoto, Japan). Production of ^15^N-labeled ZO-1(PDZ1) was achieved through on-column digestion by PreScission™ Protease followed by gel filtration chromatography, using a Superdex 75 column (Cytiva, Tokyo, Japan).

The expression and purification of the second PDZ domain of mouse LNX1p80 (residues 381–467) (LNX1(PDZ2)) were performed in similar manners. The recombinant GST-tagged LNX1(PDZ2) (^15^N-labeled) was expressed by *Escherichia coli* BL21 (*DE3*) in 1 L M9 minimal media with ^15^N-ammonium chloride as the sole nitrogen and source, supplemented with divalent cations and vitamins at 20 °C. The fusion protein was captured by affinity purification on GST-accept. Production of ^15^N-labeled LNX1(PDZ2) was achieved through on-column digestion by PreScission™ Protease, followed by gel filtration chromatography, using a Superdex 75 column.

### 2.3. NMR Titration Experiments

To examine the direct binding of the flavonoids to ZO-1(PDZ1) or LNX1(PDZ2), a series of 2D ^1^H-^15^N heteronuclear single quantum coherence (HSQC) spectra with WATERGATE water suppression [19] was recorded at 25 °C. ZO-1(PDZ1) was dissolved in 5% D_2_O–95% H_2_O, containing 20 mM MES buffer (pH 5.9). LNX1 was dissolved in 10% D_2_O–90% H_2_O, containing 20 mM MOPS, 150 mM NaCl and 50 mM MgSO4 (pH 6.8). In the titration, 0, 2, 4, 5 and 10 molar equivalences of NAR, NRG, NRT, HST, NHD, HSD, QUE and RUT were added to 0.1 mM ^15^N-labeled ZO-1(PDZ1) or LNX1(PDZ2) until obvious precipitation was observed. All the normalized chemical shift changes in the ^1^H-^15^N HSQC spectra upon ligand titration were calculated as Δδ_normalized_ = {Δδ(^1^H)^2^ + [Δδ(^15^N)/5]^2^}^1/2^, where Δδ(^1^H) and Δδ(^15^N) are chemical shift changes in amide proton and amide nitrogen. Visualization of the normalized chemical shift changes upon compound binding was performed by the program PyMOL (Schrödinger, Inc., NY, USA) [20] onto the ribbon representation of PDB code 2H3M. Each threshold value was calculated using the method developed by Schumann et al. [21].

### 2.4. Cell Culture

MDCK II cells were cultured in Dulbecco’s modified Eagle’s medium (DMEM), supplemented with 10% fetal bovine serum (FBS; Biosera, Ringmer, UK) and 1% penicillin/streptomycin (Gibco, NY, USA) at 37 °C with 5% CO_2_. Aliquots of 30 × 10^4^ cells were plated on each well of a 6-well, 35 mm plate (Corning Japan, Tokyo, Japan), or 1.0 × 10^4^ cells were plated on each well of the upper transwell filter of a Millicell 24-cell culture plate (Merck-Millipore, Darmstadt, Germany). Twenty-four hours after plating, the culture medium was changed to the medium containing 10 and 100 μM of NAR, NRG, NRT, HST, NHD, HSD, QUE, RUT and AICAR. A quantity of 10 μM LY294002 was added 3 h before harvesting. After 48 h of exposure to the compounds, the cells were subjected to immunofluorescence, cell morphology, Western blotting, TEER measurement or two-dimensional electrophoresis analysis. Cells exposed to 0.1% DMSO were used as a control.

### 2.5. Immunofluorescence Microscopy

The cells were fixed with cold 1× phosphate-buffered saline containing 4% paraformaldehyde and incubated with primary antibodies for 24 h at 4 °C. The cells were then incubated with secondary antibodies for 1 h at room temperature, with Anti-CLD-2 used as the primary antibody. The fluorescence images were obtained using fluorescence microscopy equipped with a color charge-coupled device (CCD) camera (IX-71 and DP-70, respectively; Olympus, Tokyo, Japan; scale bar: 20 μm).

### 2.6. Cell Morphology Analysis

To characterize the morphological parameters of the cell morphology, differential interference contrast (DIC) images were digitalized and analyzed by ImageJ software (National Institutes of Health). The cell edge was traced manually, and the cell area was quantified by the “analyze measure” command in the “polygon selection” menu. The longer and shorter axes were quantified in the “straight line selections” menu. From each viewing area, 10 cells were randomly chosen and analyzed. All parameters were normalized against corresponding values from the control cells.

### 2.7. Western Blotting

Aliquots of 30 × 10^4^ MDCK II cells were plated on each well of a 6-well, 35 mm plate (Corning Japan) and the culture medium (DMEM) containing 10 or 100 μM of the compounds of interest, with a final concentration of 0.1% of d_6_-DMSO added. Quantities of 10 μM LY294002 were added 3 h before being harvested. The cells were harvested after 48 h of incubation under 5% CO_2_ at 37 °C. The cells were rinsed twice with DMEM medium without serum and recovered with 100 μL of the buffer containing sodium dodecyl sulfate (SDS). The cells were disrupted by a mild sonication using Bioruptor (Cosmo Bio, Tokyo, Japan) for 4 min (duty cycle 50%). Each crude protein sample was analyzed by SDS–polyacrylamide gel electrophoresis (SDS-PAGE). The gel was washed with the buffer containing 25 mM Tris-HCl (pH 7), 192 mM glycine and 20% methanol, and electroblotted to the PVDF membrane (ATTO, Tokyo, Japan). The PVDF membrane was blocked by 5% skim milk and incubated with the primary antibodies overnight. The membrane was then washed four times and treated with the corresponding secondary antibodies. The proteins were illuminated by Chemi-Lumi One Super solution (Nacalai Tesque) and detected using the LAS-3000 mini imaging analyzer system (Fuji Film, Tokyo, Japan).

### 2.8. Transepithelial Resistance (TEER)

MDCK II cells were cultured onto a tight monolayer on the upper transwell filter of a Millicell 24-well cell culture plate (Merck-Millipore, Darmstadt, Germany). The DMEM, supplemented with 10% FBS and 1% penicillin/streptomycin, was used. The TEER of the cell monolayer was measured with an epithelial volt-ohmmeter (Millicell^®^ ERS-2, Merck-Millipore). To assess the maturation of TJ integrity, cells with stable TEER were then exposed to 100 μM of NAR, QUE or 0.1% DMSO as diluent control.

### 2.9. Two-Dimensional Electrophoresis

Aliquots of 30 × 10^4^ MDCK II cells were plated on each well of a 6-well, 35 mm plate and the culture medium (DMEM) containing 100 μM NAR or AICAR, with a final concentration of 0.1% of d6-DMSO was added. The cells were harvested after 48 h of incubation under 5% CO_2_ at 37 °C. Quantities of 10 μM LY294002 were added 3 h before being harvested. The cells were rinsed twice with DMEM medium without serum, and recovered with 500 μL of the cell lysis buffer (60 mM Tris, 5 M urea, 1 M thiourea, 1% protease inhibitor (Nacalai Tesque), 1% CHAPS, 1% TritonX-100, 1% dithiothreitol, pH 8.8). The cells were disrupted by mild sonication using Bioruptor (Cosmo Bio, Tokyo, Japan) for 5 min (duty cycle 50%). Each crude protein sample was analyzed by agar gel (ATTO) according to isoelectric point with a buffer (upper electrode solution: 0.2 M sodium hydroxide; lower electrode solution: 6 mM phosphoric acid), and the gel was fixed with trichloroacetic acid, washed with water and equilibrated with a buffer (0.5 M Tris-HCl (pH 6.8), 2% SDS, 0.001% bromophenol blue). The agar gel was then applied to the SDS-polyacrylamide gel and separated by molecular weight, followed by fixation, Coomassie Brilliant Blue staining, decolorization, and silver staining. Silver staining was performed using Silver Stain II Kit Wako, according to the manufacturer’s instruction.

### 2.10. Statistical Analysis

All values are expressed as means with their standard error of mean (SEM). Statistical analyses were performed by one-way ANOVA followed by a Tukey–Kramer test. A difference of *p* < 0.05 was considered significant.

## 3. Results

### 3.1. Changes in Cell Morphology Induced by Flavonoids

**Figure 2:**
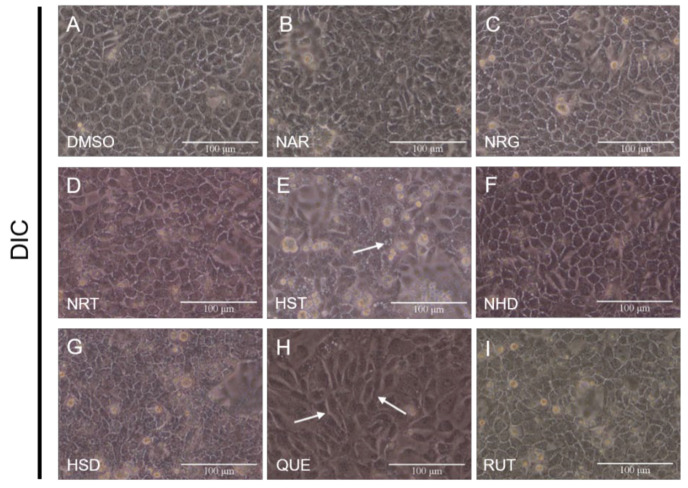
Effects of flavonoids on the morphology of Madin–Darby canine kidney (MDCK) II cells. Bright-field differential interference contrasts (DIC) for the images with corresponding flavonoids that are arrayed. Cells were treated with flavonoids at a concentration of 100 μM for 48 h (we decided this condition from the result of Supplementary Figure S2). Arrows indicate the changes. (**A**) Control (DMSO), (**B**) NAR, (**C**) NRG, (**D**) NRT, (**E**) HST, (**F**) NHD, (**G**) HSD, (**H**) QUE, and (**I**) RUT. Scale bar = 100 µm. DMSO: dimethylsulfoxide, NAR: naringenin, NRG: naringin, NRT: narirutin, HST: hesperetin, NHD: neohesperidin, HSD: hesperidin, QUE: quercetin, RUT: rutin

First, we observed morphological changes in the MDCK II cells after flavonoid exposure by bright-field phase contrast microscopy (Figure 2). In this study, MDCK II cells start to form a continuous TJs after approximately 24 h from plating. We assessed the effects of flavonoids by exposing the cells at this time point. There were no remarkable morphological changes that had been induced by the flavonoids, except HST and QUE. The cells treated with HST showed several bright granular spots surrounded by normal cells, which were glowing white. The cells treated with QUE showed a drastic morphological change, in which their morphology, normally cobblestone-like for typical epithelial cells, was transformed, becoming more slender and fibroblast-like. We recently observed a similar morphological change to a fibroblast-like elongation of MDCK II cells, when the cells were exposed to baicalein [12].

We further analyzed this morphological change quantitatively, measuring the longer and shorter axial lengths of the cells from the image for comparison (Supplementary Figure S1). We found that the cells were elongated, stretching approximately 180% toward the long axis. After 96 h of continuous QUE exposure, cell elongation reached 220% (Supplementary Figure S1, left panel). Interestingly, this morphological change appeared to be irreversible. The cells were washed with a medium without QUE for 48 h, and when they were compared with the control cells, the cell morphology did not return to normal. In addition, continuous 96-h QUE exposure induced an elongation of the shorter axis of the cells to 130%, on more than 300% of the relative cell area. It should be noted that this drastic change was not observed for RUT, a glycoside of QUE.

### 3.2. Changes in TJ Structure Induced by Flavonoids

**Figure 3:**
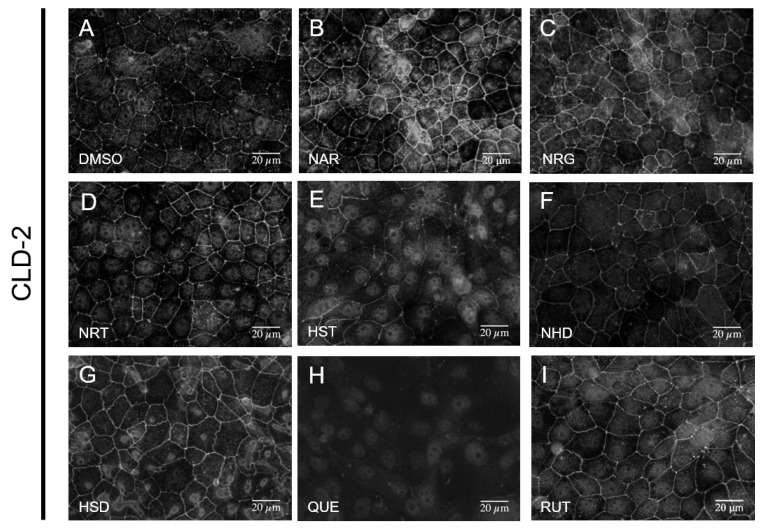
Effects of flavonoids on tight junction integrity of MDCK II cells. Immunofluorescence staining of CLD-2 images are arrayed. Cells were treated with flavonoids at a concentration of 100 μM for 48 h. (**A**) control (DMSO), (**B**) NAR, (**C**) NRG, (**D**) NRT, (**E**) HST, (**F**) NHD, (**G**) HSD, (**H**) QUE, and (**I**) RUT. Scale bar = 20 µM. Brightness is modified to 160%. MDCK: Madin–Darby canine kidney, CLD: claudin, DMSO: dimethylsulfoxide, NAR: naringenin, NRG: naringin, NRT: narirutin, HST: hesperetin, NHD: neohesperidin, HSD: hesperidin, QUE: quercetin, RUT: rutin

Subsequently, using immunofluorescent microscopic observation, we investigated the effects of the flavonoids against the TJs of MDCK II cells. For this purpose, anti-CLD-2 antibody staining was used because CLD-2 is believed to be the most abundant CLD in MDCK II cells. When exposed to 100 μM of flavonoids for 48 h, NAR, NRG and HSD exhibited enhancements of the localization of CLD-2 on the inter-cellular space of the lateral membrane (Figure 3). Conversely, the cells exposed to QUE showed reductions in the amount of localized CLD-2 at the lateral membrane. When treated with QUE, CLD-2 almost completely disappeared in the intercellular membrane compartment. Since we observed that the irreversible morphology change was induced by QUE, we further examined whether the CLD-2 recovered into the intercellular membrane space by replacement with a fresh medium after 48 h QUE exposure. In this situation, we were able to observe a partial recovery of CLD-2 localization at the lateral membrane (Supplementary Figure S3, middle). In other words, the effect of QUE against CLD-2 integration into the TJ compartment was partly reversible, whereas the effect against the cell morphology was irreversible.

**Figure 4:**
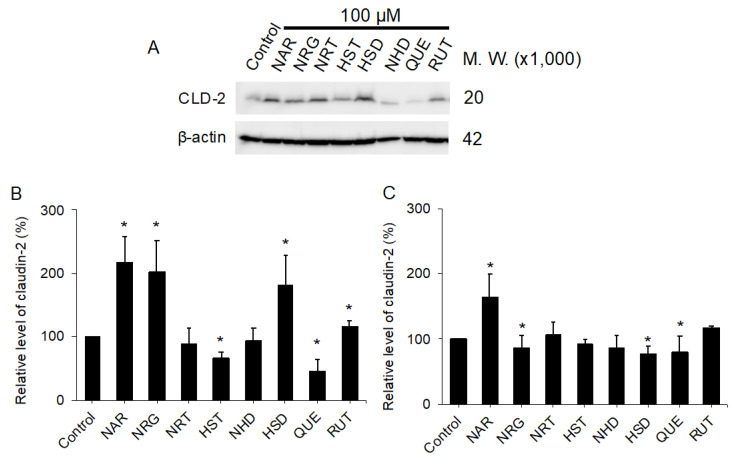
Changes in the relative amounts of protein CLD-2 after 100 or 10 µM flavonoid treatment for 48 h in MDCK II cells. (**A**) Western blotting analysis of CLD-2 expression in cell lysates from control and 100 µM flavonoid-treated MDCK II cells. (**B**,**C**) Quantitative analysis with densitometry of 100 µM (**B**) and 10 µM (**C**) flavonoid-treated MDCK II cells. Error bars indicate standard deviations. Statistical analyses were performed via Tukey–Kramer multiple comparison tests. Different from the value of the control cells, * *p* < 0.05. NAR: *n* = 16; HST, HSD, QUE: *n* = 8; NRG, NRT, NHD: *n* = 7; RUT: *n* = 3. In Supplementary Figure S5, we show the raw materials used for (**B**,**C**). MDCK: Madin–Darby canine kidney, CLD: claudin, NAR: naringenin, NRG: naringin, NRT: narirutin, HST: hesperetin, NHD: neohesperidin, HSD: hesperidin, QUE: quercetin, RUT: rutin

Accordingly, the protein levels of CLD-2 in the cells treated with the flavonoids were examined by Western blotting. Figure 4A indicated that CLD-2 extracted from the cells was exposed to 100 μM of flavonoids for 48 h, whereas Figure 4B,C show bar graphs of the normalized CLD-2 protein levels treated with 100 μM (Figure 4B) and 10 μM (Figure 4C) of indicating flavonoids. Treatment with 100 μM of flavonoids resulted in a 2.1-fold increase in NAR, a 2-fold increase in NRG, and a 1.8-fold increase in HSD, in terms of CLD-2 protein level. By contrast, QUE showed a reduction in CLD-2 protein levels at 30%. This change in CLD-2, induced by the flavonoids, seemed to result in a dose-dependent situation, since exposure to 10 μM flavonoids did not induce remarkable CLD-2 level changes except with NAR (Figure 4C). Thus, we concluded that NAR was the most potent flavonoid for increasing CLD-2 accumulation. We further analyzed the QUE-treated cells after a 48-h recovery in the medium without QUE. In Supplementary Figure S4, the Western blot analysis clearly shows that the CLD-2 protein level also recovered after the removal of QUE from the medium.

**Figure 5:**
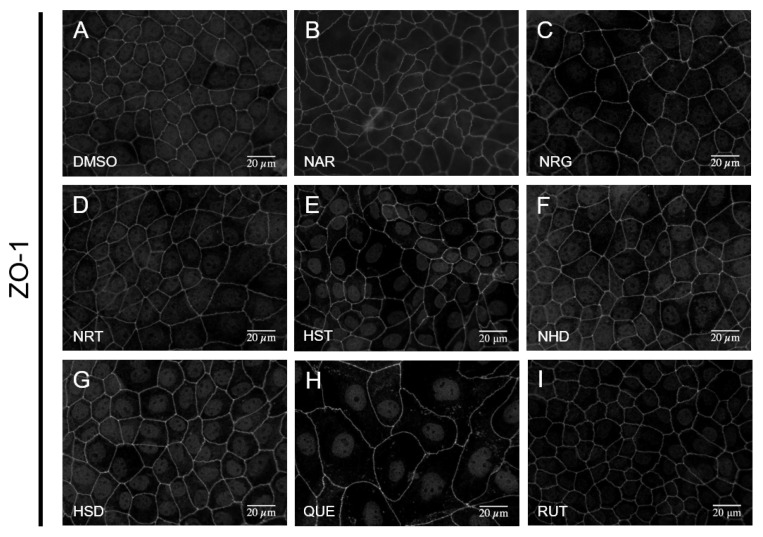
Effects of flavonoids on TJ integrity of MDCK II cells. Immunofluorescence staining of ZO-1 images are arrayed. Cells were treated with flavonoids at a concentration of 100 μM for 48 h. (**A**) control (DMSO), (**B**) NAR, (**C**) NRG, (**D**) NRT, (**E**) HST, (**F**) NHD, (**G**) HSD, (**H**) QUE, and (**I**) RUT. Scale bar = 20 µM. Brightness is modified to 160%. MDCK: Madin–Darby canine kidney, ZO-1: zonula occludens-1, DMSO: dimethylsulfoxide, NAR: naringenin, NRG: naringin, NRT: narirutin, HST: hesperetin, NHD: neohesperidin, HSD: hesperidin, QUE: quercetin, RUT: rutin

We also analyzed the effect of the flavonoids against the TJ scaffold protein ZO-1. ZO-1 is known as one of the most important organizers of the TJ [9,10], with a lack of accumulation of ZO-1 at the TJ site inducing disintegration of the TJ [22]. We observed the subcellular localization of ZO-1 on each of the flavonoid-treated MDCK II cells by immunofluorescent microscopy. Regardless of the increase (NAR, NRG, HSD) or decrease (QUE) in the amount of TJ-accumulated CLD-2, the amount of ZO-1 localized to the lateral membrane of MDCK II cells did not change (Figure 5). This suggests that either enhancement or downregulation of CLD-2 accumulation, induced by flavonoids, were independent of ZO-1, regardless of the protein’s expression level or the subcellular localization. In addition, we observed a cell swelling-like morphology in the MDCK II cells, which were exposed to QUE (Figure 5H). In the light field microscopy, the cells treated with QUE showed an elongated slender fibroblast-like shape. The cell swelling induced by QUE may be due to the rearrangement of the actin cytoskeleton, and seems to lose contractile force. Further studies on QUE toxicity for MDCK II cells are needed.

### 3.3. Assessment of Direct Interactions of the Flavonoids with either LNX1(PDZ2) or ZO-1(PDZ1)

In our previous study, we succeeded in determining the solution structure of mouse ZO-1(PDZ1) by NMR and demonstrated the mutually competitive binding of CLD-3 C-terminal peptide and phosphatidylinositol phosphate [23]. Accordingly, a mechanism that directly binds small ligands to the canonical peptide binding pocket of ZO-1(PDZ1) could reduce epithelial TJ integrity [12]. On the other hand, LNX1p80 is one of the known CLDs-targeted E3 ubiquitination enzymes responsible for TJ downregulation, making it possible that LNX1(PDZ2) is responsible for this process (the direct binding of a peptide derived from CLD-1 and LNX1(PDZ2) was confirmed by the similar NMR titration experiments (Supplementary Figure S6H)). Thus, compounds that bind LNX1(PDZ2) may increase epithelial TJ integrity by accumulating CLDs at cell boundaries. For our purposes, we assessed the presence or absence of direct interactions between these PDZ domains and the flavonoids via the solution NMR technique.

At the presence of 2 equivalents of HST, 5 equivalents of NRG or HSD, or 10 equivalents of NAR, NRT or NHD, small but substantial chemical shift perturbations (CSPs) were observed in the HSQC spectra of ^15^N-labelled ZO-1(PDZ1) (Supplementary Figure S7). The key residues that showed remarkable CSPs were analyzed, and were surrounded, in all cases, by the residues that form the canonical CLD binding pocket of ZO-1(PDZ1). QUE precipitated ZO-1(PDZ1) by adding four equivalents. These data suggested the direct interaction between ZO-1(PDZ1) and the following flavonoids: NRG, HSD, NAR, NRT, NHD, HST, and QUE. Among them, the affinity of HST and QUE to ZO-1(PDZ1) might be stronger than the others. Thus, the TJ-mitigating activity of HST and QUE can be partly explained by an inhibition of ZO-1. Of course, the contribution of the other signaling process must not be ruled out. In contrast, we did not observe any remarkable CSPs when LNX1(PDZ2) was titrated by NRG, NAR and HSD (Supplementary Figure S6). All these weak molecular interactions of flavonoids with the corresponding PDZ domains of the TJ-regulating cytosolic proteins are summarized in Supplementary Table S1. Based on this observation, we conclude that the CLD-2-accumulating activity of NRG, NAR and HSD was not due to the inhibition of LNX1p80.

### 3.4. Assessment of Change in the Functional TJ Integrity Induced by the Flavonoids

**Figure 6:**
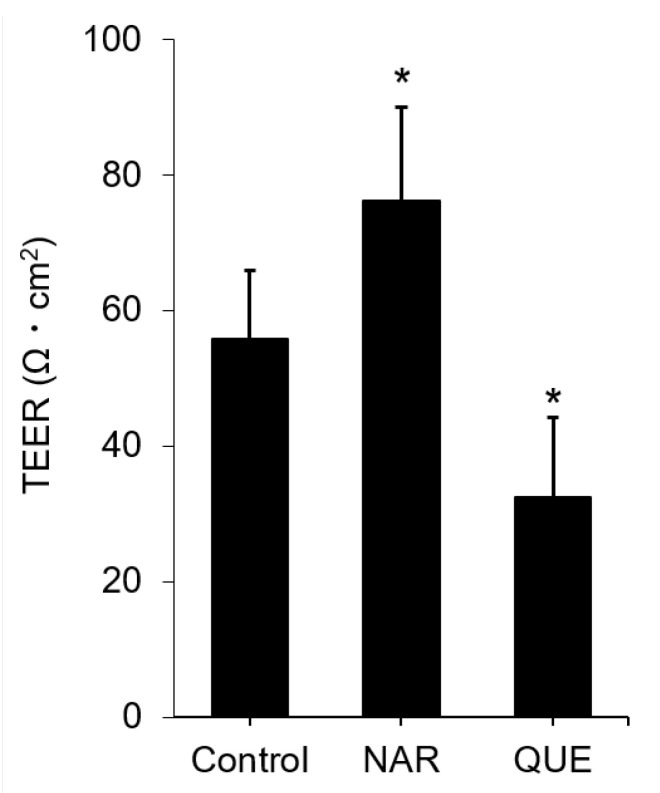
Effects of NAR and QUE on TEER in MDCK II cells. Cells were treated with the flavonoids (NAR or QUE) at a concentration of 100 μM for 48 h. Error bars indicate standard deviations. Statistical analyses were performed by Turkey–Kramer multiple comparison tests. Different from the value of the control cells, * *p* < 0.05. NAR: *n* = 5; QUE: *n* = 3. MDCK: Madin–Darby canine kidney, NAR: naringenin, QUE: quercetin, RUT: rutin, TEER: transepithelial electrical resistance.

Next, we assessed whether the significant CLD-2 accumulation by NAR or the CLD-2 disintegration by QUE were related to TJ function. For this purpose, we employed the measurement of transepithelial electrical resistance (TEER). As a result, NAR showed an approximately 30% increase in TEER compared to that of the control cells treated with 0.1% DMSO (Figure 6). Similarly, QUE exhibited a 40% reduction in TEER. Since CLD-2 is the channel-forming “leaky” CLD that can transport water and cations, this observation seemed controversial. Thus, we investigated the changes on the other CLDs, CLD-1, -3, -4 and -7, by a similar method (Supplementary Figure S8). We found that CLD-4 changed to the opposite direction, whereas the changes of CLD-1, -3 and -7 were weakly correlated with the CLD-2 protein expression level in the NAR-treated MDCK II cells. The remarkable increases in cellular CLD-2 level (Figure 4) and the TJ-accumulated CLD-2 (Figure 3) are seemingly consistent with in vitro TJ functionality as assessed by TEER. Accordingly, the NAR-induced changes in the other CLDs were smaller than that in CLD-2. Nevertheless, the effect of flavonoids on TEER is likely related to the modulation of the expression patterns of not only CLD-2 but also various CLDs. It is still not clear whether these smaller fluctuations in various CLDs were directed by the flavonoids or were indirect effects induced by CLD-2 alteration. The changes in the other TJ components have not been examined. Thus, further study is needed.

### 3.5. Comparison of Known TJ Integrity Enhancers

**Figure 7:**
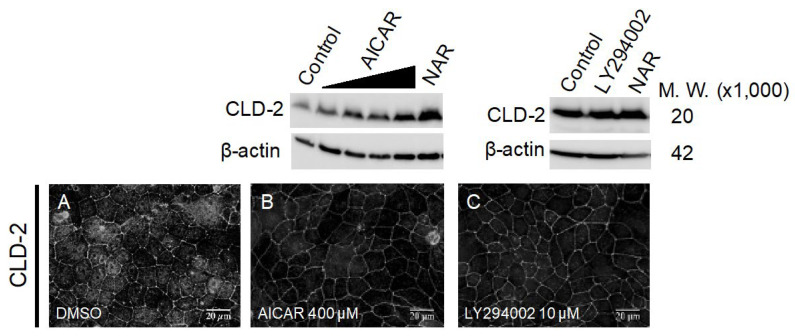
Effects of AICAR and LY294002 on CLD-2 level and localization in MDCK II cells. Top panel: Western blotting analysis of CLD-2 level in cell lysates from control and 100 µM NAR-treated, 20, 100, 200 and 400 µM AICAR-treated, and 10 µM LY294002-treated MDCK II cells. Bottom panel: Immunofluorescence staining of CLD-2 images are displayed. Cells were treated with AICAR at a concentration of 400 µM for 48 h (**B**) or LY294002 at a concentration of 10 µM for 3 h (**C**), and DMSO is used for control (**A**). Scale bar = 20 µM. Brightness is modified to 130%. In Supplementary Figure S9, we show raw materials for membrane. M.W.: molecular weight, CLD: claudin, MDCK: Madin–Darby canine kidney, DMSO: dimethylsulfoxide, NAR: naringenin, AICAR: 5-aminoimidazole-4-carboxamide-1-β-d-ribofuranoside

As shown above, we found that NAR is the strongest TJ-enhancing flavonoid among the examined eight flavonoids. Accordingly, we compared the TJ-enhancing activities of NAR to those of known TJ-enhancing reagents, 5-aminoimidazole-4-carboxamide- 1-β-D-ribofuranoside (AICAR) and LY294002. AICAR is a nucleotide analogue and an activator of AMPK [24]. In some epithelial cell lines, it is reported that activation of the AMPK signaling pathway promotes biogenesis of the TJs, thereby enhancing TJ integrity [24,25]. It is important to note that AMPK is temporally activated during TJ biogenesis in MDCK II cells, when induced by the Ca^2+^ switch experiment [26]. Though it should also be noted that the PI3K pathway is known as a negative regulator of TJ integrity in some epithelial cells. For example, in MDCK II cells, oxygen stress caused the TJs’ attenuation in a PI3K pathway-dependent manner [27]. Meanwhile, LY294002, an inhibitor of PI3K, prevented the TJ downregulation induced by oxidative stress [27]. This aligns with our recent demonstration that phosphatidylinositol triphosphate, a product of PI3K, directly binds ZO-1(PDZ1) at the apicolateral membrane. We hypothesized that phosphatidylinositol triphosphate may functionally compete with CLDs to bind ZO-1, thereby suppressing the TJ as a physiological negative regulator [23]. In fact, we observed a clear accumulation of CLD-2 in the TJ compartment of LY2940002-treated MDCK II cells, regardless of oxygen stress conditions (Figure 7, Supplementary Figure S9). We compared the TJ-enhancing activity of NAR with that of AICAR and LY294002. When the cells were treated with 400 μM of AICAR for 48 h, the accumulation of CLD-2 at a similar level was observed on the cells exposed to 100 μM NAR, and the protein level of CLD-2 became fairly similar. In addition, we observed that, with a treatment of 10 μM of LY294002 for 3 h, there was a moderate but increasingly fast accumulation of CLD-2 in the TJ compartment.

### 3.6. Comparison of the Expressed Protein Levels in MDCK II Cells Treated with NAR, AICAR and LY294002 by Whole-Cell 2D-PAGE Analysis

**Figure 8:**
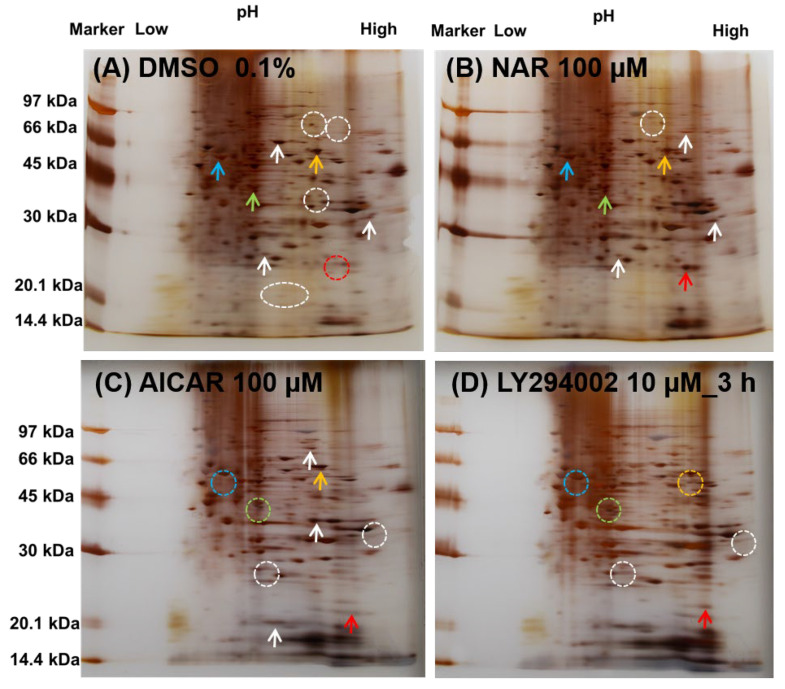
Protein expression profiling of NAR-, AICAR- and LY294002-treated MDCK II cells. Cells were treated with NAR (100 µM) (**B**) or AICAR (100 µM) for 48 h (**C**) or LY294002 (10 µM) for 3 h (**D**) and DMSO is used for control (**A**). Decreased or vanished protein spots were marked with broken line circles, and increased spots were marked with arrows. Red: weak at DMSO, and increased at NAR, AICAR and LY294002; orange: weak at DMSO and LY294002, and increased at NAR and AICAR; pea green and sky blue: increased at DMSO and NAR, and vanished at AICAR and LY294002. The predicted candidate of each protein is summarized in Table 1. MDCK: Madin–Darby canine kidney, DMSO: dimethylsulfoxide, NAR: naringenin, AICAR: 5-aminoimidazole-4-carboxamide-1-β-d-ribofuranoside

Finally, the signaling mechanisms underlying the compound-induced TJ-integrity enhancement in MDCK II cells were studied. We observed similar levels of increased CLD-2 accumulation in the TJ compartment when the cells were treated with either 100 μM NAR, 400 μM AICAR or 10 μM LY294002. Next, we intended to examine the changes in the expression levels of the other proteins. For this purpose, we employed whole-cell two-dimensional polyacrylamide electrophoresis (2D-PAGE) analysis (Figure 8). The whole-cell protein extracts, from either NAR-, AICAR- or LY294002-treated MDCK II cells, were analyzed and compared with those of the DMSO-treated cell as a negative control. We found several protein spots showing changes. Several spots with significant changes, whether in their intensity or mobility, are marked by arrows in Figure 8.

In all three compound-treated cells, there was an increased intensity of the spot at approximately 24.5 kDa and pI 8.47 (red arrow), compared to the control (Table 1). We determined this spot to be CLD-2, by its theoretical molecular weight (M.W.) and isoelectric point (pI). However, we observed many different changes of spots among the three compounds, indicated by different-colored arrows (white, orange, pea green, and sky blue). For example, the spot at approximately 64.0 kDa and pI 8.32, identified by the orange arrow, was observed in the control and the NAR- and AICAR-treated cells, whereas the corresponding spot migrated in the LY294002-treated cell. Similarly, the spots identified by the sky blue and pea green arrows were only observed in the control and NAR-treated cells, whereas the spots in the AICAR- and LY294002-treated cells completely migrated. These spots were tentatively assigned as AMPKα, PPARA and MAPK1, by their theoretical molecular weights and the calculated pI. In addition, we marked with white arrows several protein spots that were differently observed in the four panels, though these spots had not yet been identified. Taking all these differences into account, we concluded that the signaling pathway that promotes the enhancement of some CLDs by NAR is driven by a yet-unknown signaling pathway other than the AMPK or the PI3K pathways.

## 4. Discussion

In this study, we examined the TJ-modulating activity of eight flavonoids classified into three groups. The groups were represented by their aglycones, NAR, HST and QUE, with or without different sugar moieties, neohesperidose (NRG and NHD) and rutinose (NRT, HSD, and RUT). Among them, three flavonoids (NAR, NRG and HSD) exhibited an increase in CLD-2 accumulated in the TJ compartment, whereas QUE showed a reduction in CLD-2. Accordingly, the TEER of the cell monolayer treated by NAR and QUE showed an increase and decrease, respectively. For the NAR treatment, some other CLDs (CLD-1,-3 and -7) correlatively increased (Supplementary Figure S8). The rest of the three flavonoids, NRT, NHD and RUT, did not show any remarkable effect on the TJs, those located on the MDCK II cells, at the concentration range used in this study. This observation about QUE seems somehow contradictory to the reported observation, in which QUE increased CLD-2 and/or the other CLDs. It should be noted that QUE is known as a TJ integrity enhancer when exposed for a short time [28]. However, several reports suggest QUE may increase the bioavailability of several drugs as a result of drug-to-drug interactions [29]. In this study, our result is inconsistent with the reported QUE activity since QUE normally increases TJ integrity. Different cellular responses against QUE treatment are assumed, and these may depend on different cell types. Some remarkable differences between the existing reports and this study are summarized in Supplementary Table S2. Given the pharmacological importance of QUE, further investigation is needed. Surprisingly, we observed the QUE-induced morphological change of the cobblestone-like epithelial phonotype into a slender fibroblast-like cell morphology (Figure 2, middle bottom panel). We recently reported similar changes in the morphology of the MDCK II cells, which were exposed to high-dose baicalein [12]. In the case of baicalein, we demonstrated the involvement of the transforming growth factor-β (TGFβ) signaling pathway. Similarly, there are some reports that QUE may induce TGFβ expression or enhance TGFβ signaling [30,31,32]. However, since there are also some controversial reports that QUE inhibits TGFβ signaling in various cells [33,34,35], the mechanism of QUE-induced morphological change remains unclear. The molecular mechanisms of QUE-induced cell morphology change and its relationship with the adverse effect of QUE should be further studied.

CLD-2 is expressed in several normal tissues, including the small intestine and renal proximal tubule. CLD-2 was shown to work as the channel-forming CLD that mediates the paracellular transport of water and cations [36]. In this study, we observed a positive correlation between the protein expression level of CLD-2 and the TEER of the MDCK II cell monolayer. This seems inconsistent with the known fact that CLD-2 is believed to be a CLD of high water- and cation-permeability. This time, we mainly focused on CLD-2, but we also observed some changes in the other CLDs. Thus, we assumed that the amount of CLD-2 may regulate either the amount or the integrity of other CLDs that are modulated by the flavonoids.

When focusing on NAR- and QUE-series flavonoids, aglycones (NAR and QUE) consistently showed stronger pharmacological effects than the others, although the direction of the activity runs in the opposite direction (TJ enhancement and TJ mitigation). While NRG (neohesperidoside) showed mild activity, NRT and RUT, which are flavone-retinoids, showed no activity. The order of such pharmacological activity seems consistent with their calculated octanol–water partition coefficient (log P). For example, the XLogP3-aa values [37] of NAR and QUE are 2.4 and 1.5, respectively, showing themselves to be highly hydrophobic. In contrast, those of NRT and RUT are −1.1 and −1.3, respectively, which suggest the molecules are highly hydrophilic (the XLogP3-aa value of NRG is −0.5). Thus, we assumed that the order of TJ-modulating activity reflects the pharmacological activity of the individual cytosolic targets (for TJ enhancement and TJ mitigation) with varying bioavailability values corresponding to their hydrophobicity.

The situation of the HST-series flavonoids (HST, NHD and HSD) is quite different. The aglycone HST exhibited a weak CLD-2-reduction activity, whereas HSD showed an exceptional CLD2-accumulation activity. Judging from the XLogP3-aa value of HSD (−1.5), the molecule is not likely permeable. We assume that the target molecule is an extracellular protein. Note that a G-protein-coupled receptor, the adenosine receptor, is capable of being stimulated by HSD and promoting an anxiolytic effect. Thus, the adenosine receptor is one of the pharmacological target candidates of HSD—although the relation between the adenosine receptor and the modulation of TJ integrity requires further studies [38].

Based on these observations, we hypothesized that NAR acts to enhance TJ integrity via a cytosolic signaling pathway, and we had intended to search for a cytosolic target of NAR. First, we examined the direct interaction of the NAR with PDZ domains related to TJ homeostasis, the first PDZ domain of ZO-1(ZO-1(PDZ1)) and the second PDZ domain of LNX1 (LNX1(PDZ2)). Interestingly, a weak but specific molecular interaction between NAR and ZO-1(PDZ1) was observed in an in vitro NMR titration experiment (Supplementary Figure S7), whereas no direct interaction between NAR and LNX1(PDZ2) was observed (Supplementary Figure S6). Therefore, it is highly unlikely that the enhancement of the TJs by NAR was caused by the prolonged lifetime of CLDs via inhibition of LNX1, which is an E3-ubiquitinating enzyme specifically involved in TJ downregulation.

Next, we assessed whether the other known cellular pathways of TJ integrity enhancement contributed to NAR-induced TJ changes. To date, some possible signaling pathways that are either activated or inhibited by flavonoids have been extensively studied, though the details for most of these mechanisms—for the TJ and barrier function regulation—are still unclear. There are several reports that NAR exhibits either a protective or recovery-promoting effect on intestinal cells [13,14,16,39,40]. Further, in non-epithelial cells, NAR is known as an AMPK activator [41,42]. In other reports, NAR has shown to possibly inhibit PI3K/Akt pathways [43,44]; however, the action of NAR seems to be both stimulating and controversial [45]. In this study, we focused on the contribution of AMPK and PI3K to the NAR-induced enhancement of TJ integrity, since these possibilities are easy to assess through the use of popular compounds AICAR (AMPK activator) and LY294002 (PI3K inhibitor). Surprisingly, in our semi-quantitative whole-cell-level protein expression analysis, the protein levels induced by NAR, AICAR and LY294002 were all different. Thus, we concluded that the intracellular target pathway of NAR is unique to either the AMPK-dependent or the PI3K-dependent pathways. NAR has also been reported to be involved in an increased expression of CLDs via the activation of peroxisome proliferator-activated receptor (PPAR) degradation [45,46], those of the NF-κB component p65 [47,48] or other pathways. It is likely that NAR-induced TJ integrity enhancement is promoted by the multiple contributions of various signaling pathways.

Finally, we would like to point out that at least NAR exhibited a TJ-enhancing activity, with the enhanced accumulation of CLD-2 and some other CLDs. NRG and HSD, the other popular fruit-derived flavonoids, also showed similar CLD-2-increasing activity. Considering the barrier-enhancing activity, as several groups previously reported, together with the other beneficial effects of citrus flavonoids, such as their anti-oxidant and anti-inflammatory benefits [49], we expect the usage of NAR-rich plant extract to be beneficial as a prophylactic drug for certain inflammation-related diseases associated with disruption of the TJ. These are conditions that include Crohn’s disease, NSAIDs-induced colitis, and non-Celiac gluten sensitivity syndrome. In contrast, our results are the first demonstration of the TJ-enhancing activity of NAR for MDCK II cells. Thus, the TJ-enhancing activity of NAR in relation to the gastrointestinal epithelia, including Caco-2 cells, seems a rather common activity for various types of epithelial cells. With that in mind, the application of these food-derived flavonoids not only on gastrointestinal but also other organs, and skin, becomes very intriguing.

## 5. Conclusions

We systematically examined eight flavonoids, including NAR, HST and QUE, and their glycosides of neohesperidose (NRG and NHD) and rutinose (NRT, HSD, and RUT), using the model epithelial cell system MDCK II. We estimated the subcellular accumulation of CLD-2 in the apicolateral intercellular junctional compartment, and its expression level. We observed the CLD-increasing activity of NAR, NRG and HSD, whereas we also observed the CLD-decreasing activity of HST and QUE. Although activation of the AMPK signaling pathway is a well-known mechanism in the barrier protection activities of many flavonoids, our protein expression profile analysis suggested that NAR may have a distinct cytosolic target.

## Figures and Tables

**Table 1:** Molecular weights, isoelectric points and colored arrows in Figure 8 of proteins that are related to TJ homeostasis. CLD: claudin; AMPKα: 5′ adenosine monophosphate (AMP)-activated protein kinase; MAPK1: mitogen-activated protein kinase; PPARA: peroxisome proliferator-activated receptor alpha; PI3KCA: phosphoinositide 3-kinase catalytic subunit α; ZO-1: zonula occludens.

Protein	Molecular Weight (×1000 kDa)	Isoelectric Point	Color in Figure 8
CLD-2	24.5	8.47	red
AMPKα	64.0	8.32	orange
MAPK1	41.4	6.50	pea green
PPARA	52.2	5.86	sky blue
PIK3CA	124.3	6.88	(not shown)
ZO-1	195.5	6.24	(not shown)

## Supplementary Materials

The following are available online at https://www.mdpi.com/2072-6643/12/11/3285/s1, Figure S1. Effects of quercetin (QUE) on cell morphology in MDCK II cells, Figure S2. Effects of flavonoids on cell viability in MDCK II cells, Figure S3: Effects of flavonoids on the morphology and TJ integrity of MDCK II cells, Figure S4. Effects of QUE on CLD-2 expression in MDCK II cells. Figure S5: Effects of flavonoids on CLD-2 expression in MDCK II cells, Figure S6: Direct interaction between LNX-1(PDZ2) and the flavonoids, Figure S7: Direct interaction between ZO-1(PDZ1) and the flavonoids, Figure S8: Effects of NAR on CLD-1, -3, -4 and -7 expression in MDCK II cells, Figure S9: Effects of compounds on CLD-2 expression in MDCK II cells, Table S1: Effects of flavonoids on NMR and amount of CLD-2. Result of research is the above, Table S2: Effects of quercetin (QUE) on some CLDs. Result of research is the above.
