# Supplementary material for: Opposing Effect of Naringenin and Quercetin on the Junctional Compartment of MDCK II Cells to Modulate the Tight Junction
Source: Nutrients. 2020 Oct 27;12(11):3285. doi: 10.3390/nu12113285 (PMC7693399; doi:10.3390/nu12113285)
Supplement: Supplementary file 1 [file nutrients-12-03285-s001.pdf]

Supplementary materials.

Opposing effect of naringenin and quercetin on the junctional compartment of MDCK II cells to modulate the tight junction.

Mio Nakashima[1,2], Misaki Hisada[1,#], Natsuko Goda[1], Takeshi Tenno[1,3], Ayaka Kotake [4], Yuko Inotsume[4], Ikuo Kameoka[4], and Hidekazu Hiroaki[1,2,3,*]

[1]Graduate School of Pharmaceutical Sciences, Nagoya University, Furocho, Chikusa, Nagoya, Aichi, 464-8601, Japan, [2]Department of Biological Sciences, Faculty of Science, Nagoya University, [3]BeCerllBar, LLC., Nagoya, Aichi, Japan, [4]Cosmetics Research Department, Nicca Chemical Co. Ltd., Fukui, Japan.

Working title: *Opposing effects of naringenin and quercetin on the tight junction*

[#]Present address, Research Center, Koken Co.,Ltd., 2-13-10, Ukima, Kita-ku, Tokyo, 115-0051, Japan

*All correspondence should be addressed to Hidekazu Hiroaki, Graduate School of Pharmaceutical Sciences, Nagoya University, Furocho, Chikusa, Nagoya, Aichi, 464-8601, Japan; hiroaki.hidekazu@f.mbox.nagoya-u.ac.jp; Tel. +81(52)789-4535; Fax. +81(52)747-6816

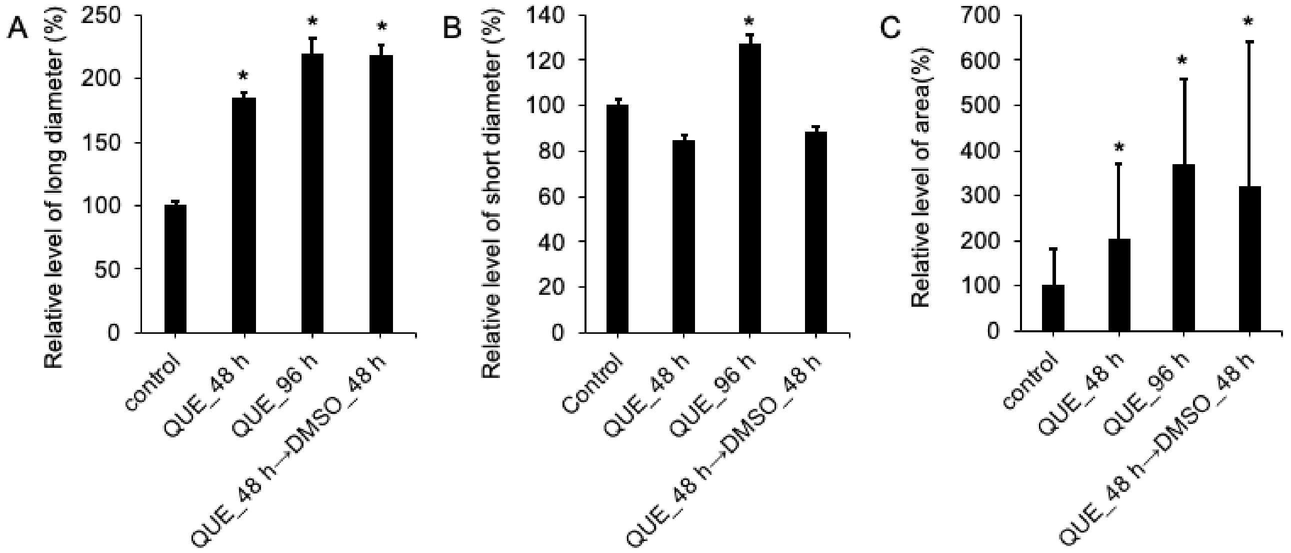

Supplementary Figure S1. Effects of quercetin (QUE) on cell morphology in MDCK II cells.

Statistical analyses were performed by Turkey-Kramer multiple comparison tests. (A) Relative level of long diameter, (B) short diameter and (C) area. Different from the value of the control cells, *p<0.05. n=11.

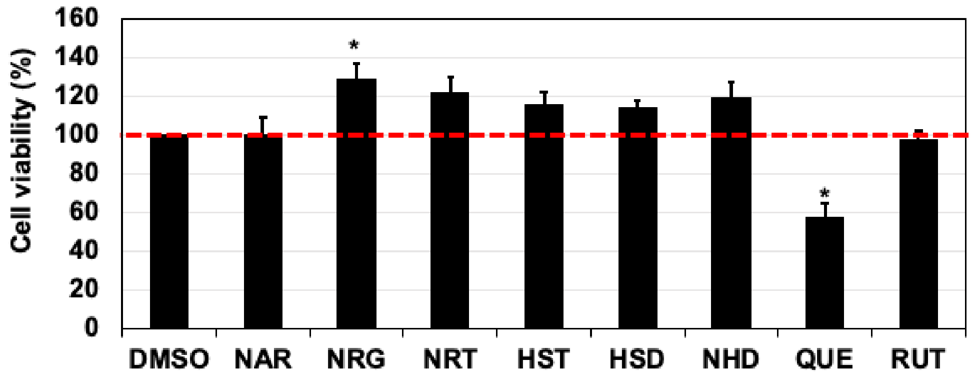

Supplementary Figure S2. Effects of flavonoids on cell viability in MDCK II cells.

Cells were treated with flavonoids at a concentration of 100 μM for 48 hours. Different from the value of the control cells, *p<0.05.

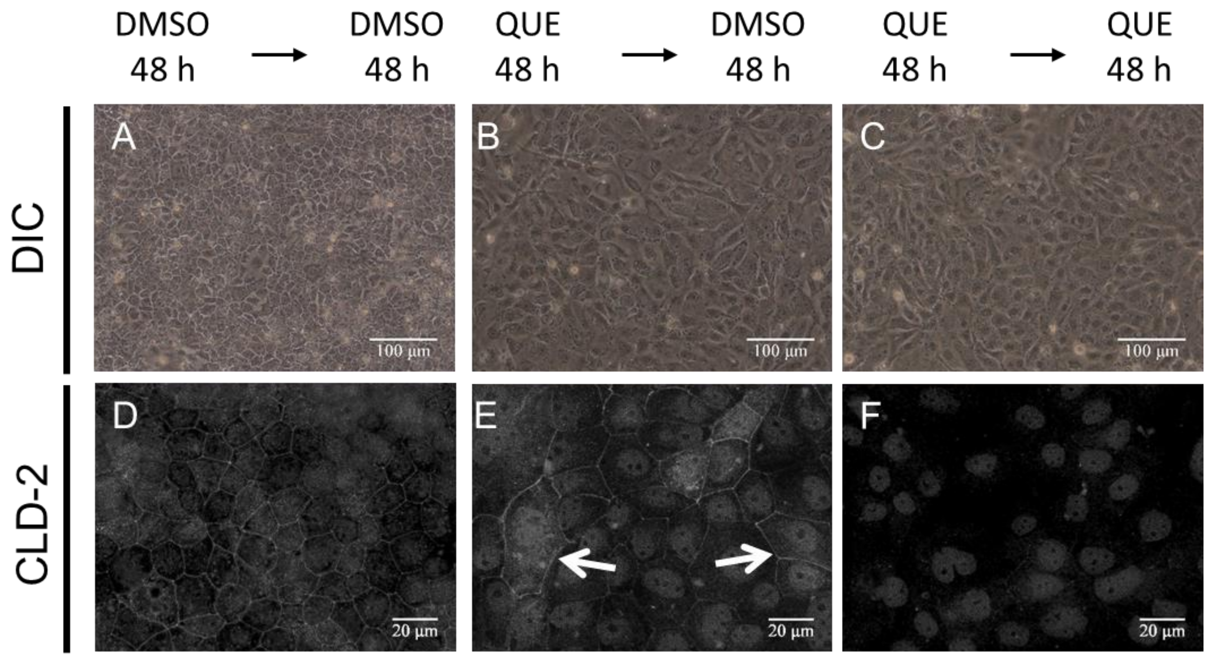

Supplementary Figure S3. Effects of flavonoids on the morphology and TJ integrity of MDCK II cells. Bright-field differential interference contrast (DIC) images (A-C) and immunofluorescence staining of CLD-2 images (D-F) of MDCK II cells are arrayed. Cells were treated with QUE at a concentration of 100 µM for 48 hours and then treated with QUE or DMSO at a concentration of 100 µM for 48 hours after washed with medium. (A, D) control (DMSO), (B, E) QUE-DMSO and (C, F) QUE-QUE. Scale bar = 100 µm. For immunofluorescence staining, brightness is modified to 150%.

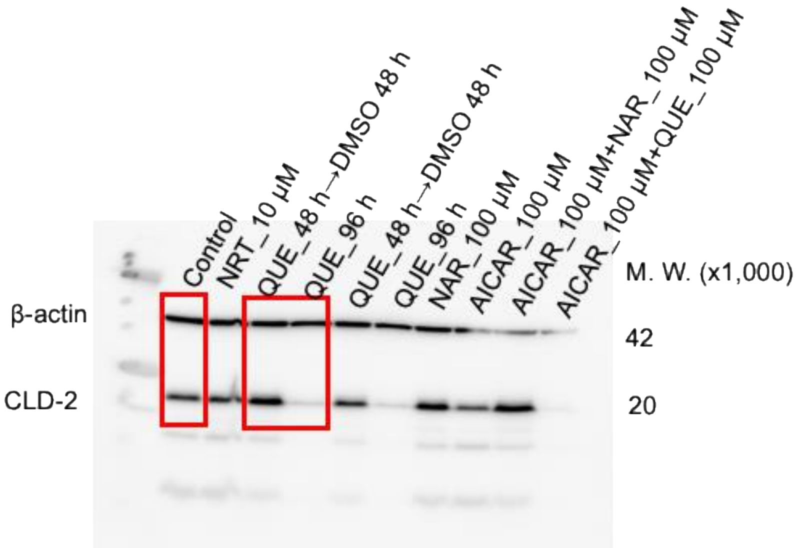

Supplementary Figure S4. Effects of QUE on CLD-2 expression in MDCK II cells.

Cells were treated with QUE at a concentration of 100 μM for 48 hours and treated with QUE or DMSO at a concentration of 100 μM for 48 hours after being washed with medium. Bands in the red line are focused.

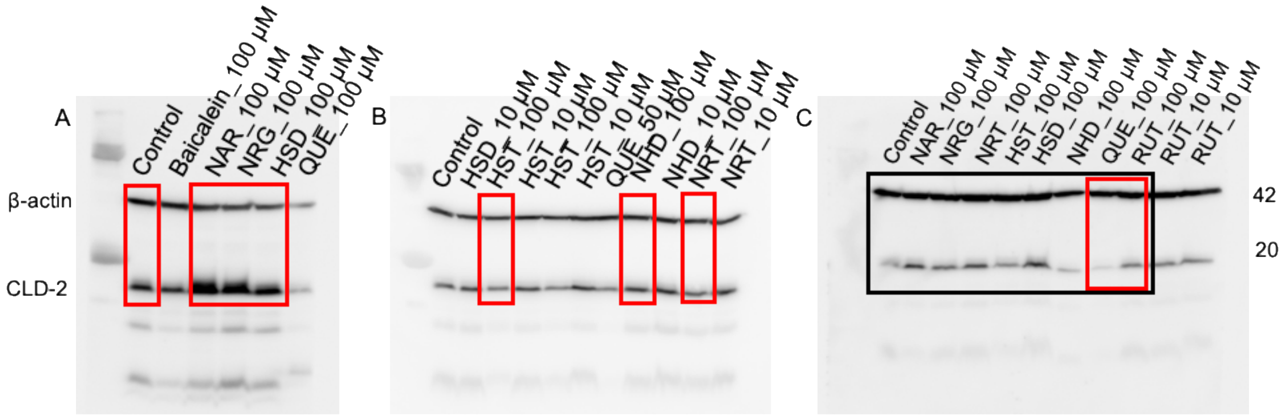

D

	Control	NAR	NRG	NRT	HST	NHD	HSD	QUE	RUT
1	100	71	49	54	91	62	59	66	108
2	100	742	300	158	88	181	190	11	110
3	100	368	351	49	21	53	456	165	133
4	100	122	135	103	52	115	132	50	
5	100	134	276	10	59	22	298	4	
6	100	147	104	186	77	128	66	13	
7	100	213	255	68	80	100	145	28	
8	100	166			70		110	26	
9	100	240							
10	100	118							
11	100	194							
12	100	343							
13	100	149							
14	100	213							
15	100	75							
16	100	195							
average	100	218	210	90	67	94	182	45	117

Supplementary Figure S5. Effects of flavonoids on CLD-2 expression in MDCK II cells.

Cells were treated with flavonoids at a concentration of 100 µM for 48 hours. (A)-(C) Bands in the red line are focused. Bands in the black line is shown in Fig. 4. (D) Value in the red line represent relative revel of CLD-2 (bands in the red line).

(A) LNX1-NAR

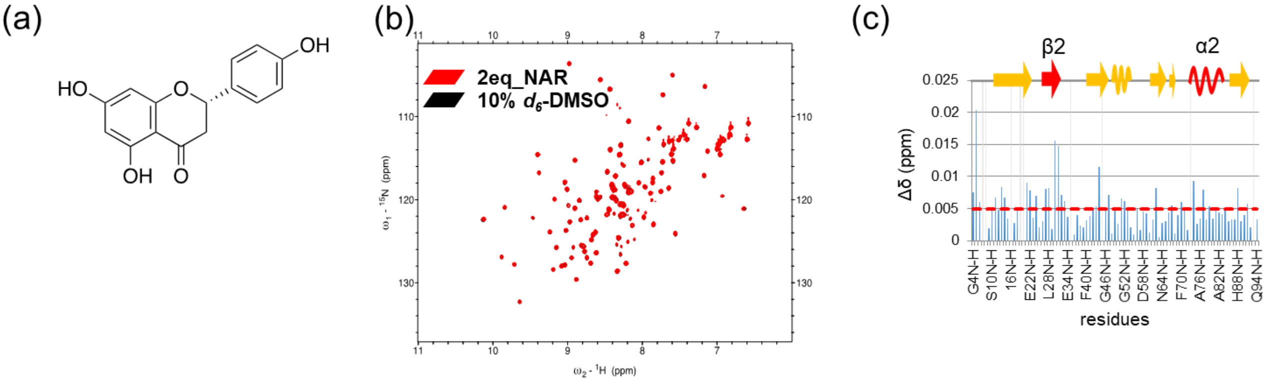

(B) LNX1-NRG

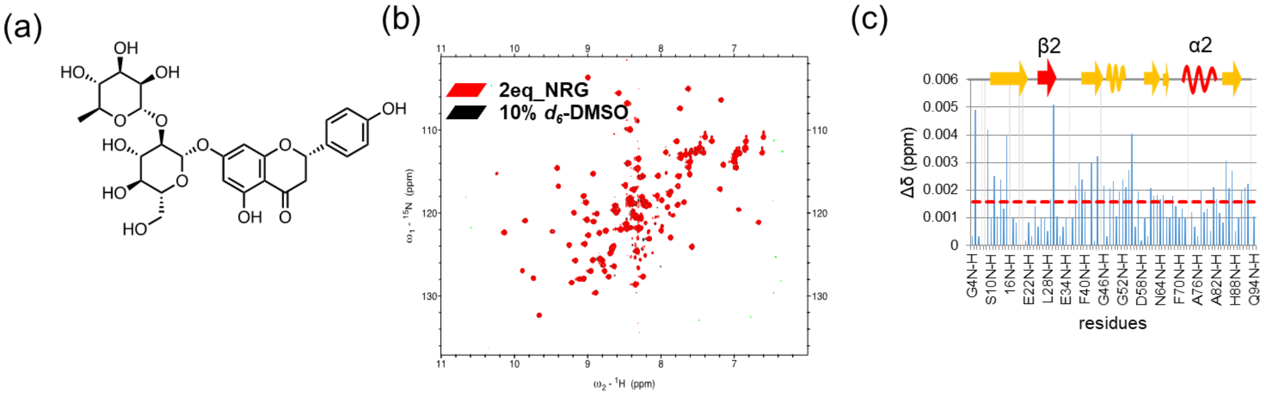

(C) LNX1-NRT

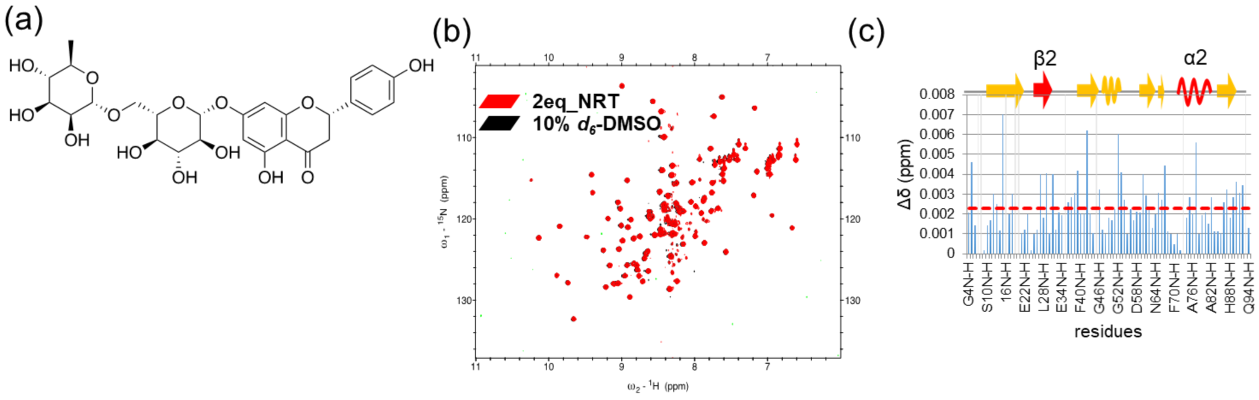

(D) LNX1-HST

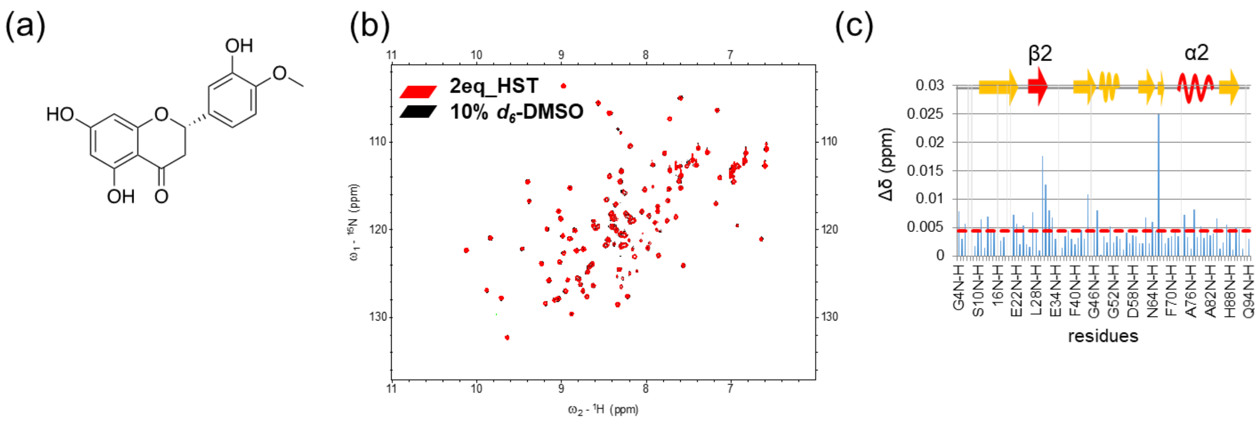

(E) LNX1-NHD

(a)

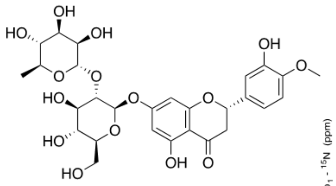

(b)

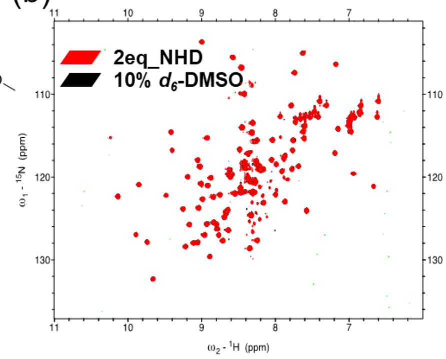

(c)

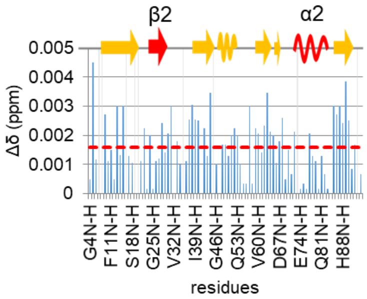

(F) LNX1-HSD

(a)

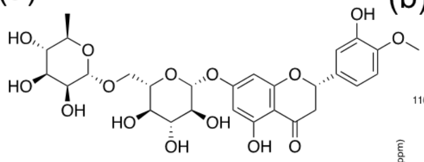

(b)

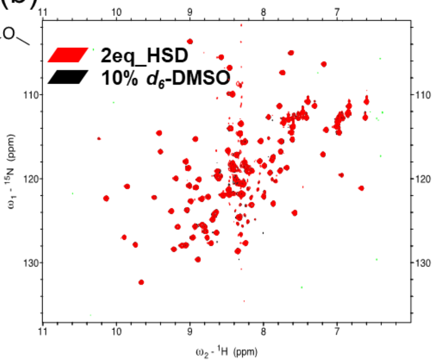

(c)

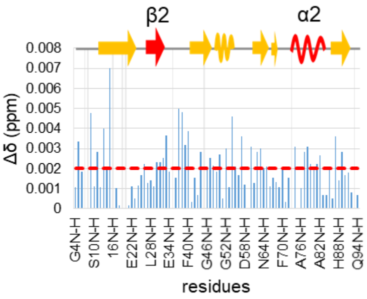

(G) LNX1-QUE

(a)

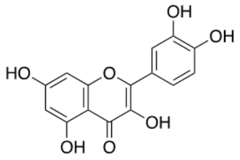

(b)

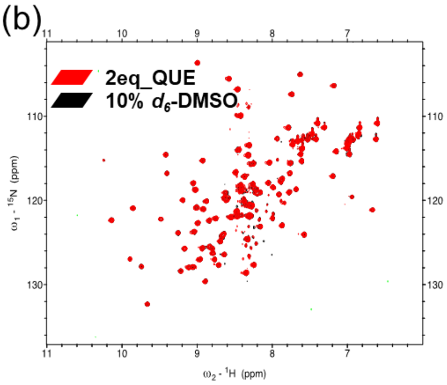

(c)

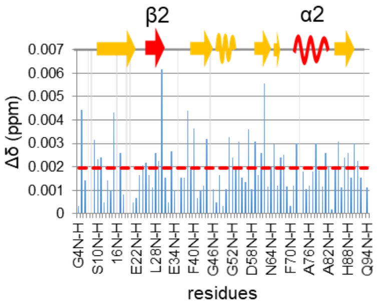

(H) LNX1-CLD1

(a)

H_2N-PTPSSGKDYV-COOH

(b)

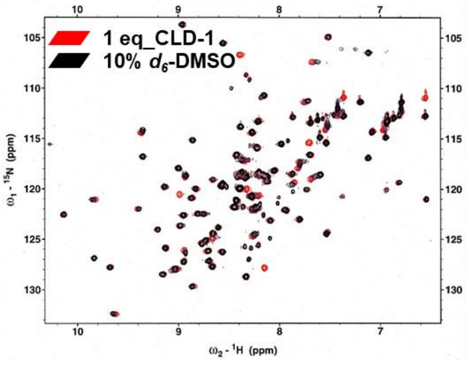

(c)

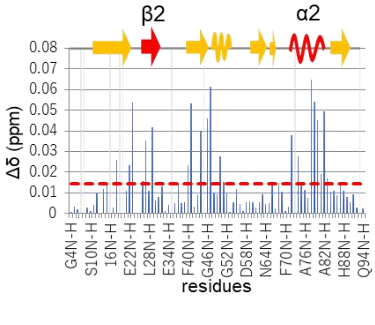

Supplementary Figure S6. Direct interaction between LNX-1(PDZ2) and the flavonoids or CLD-1. (a) (A)-(G) Chemical structure of the flavonoids titrated. (H) CLD-1 ligand. (b) Overlaid HSQC spectra of 0.1 mM LNX-1(PDZ2) in the absence (black) and presence (red) of 2 equivalent of NAR (A), NRG (B), NRT (C), HST (D), NHD (E), HSD (F), QUE (G) and 1 equivalent of CLD-1 (H). (c) Normalized chemical shift changes in the presence of 2 equivalent of NAR (A), NRG (B), NRT (C), HST (D), NHD (E), HSD (F), QUE (G) and 1 equivalent of CLD-1 (H).

(A) ZO1-NAR

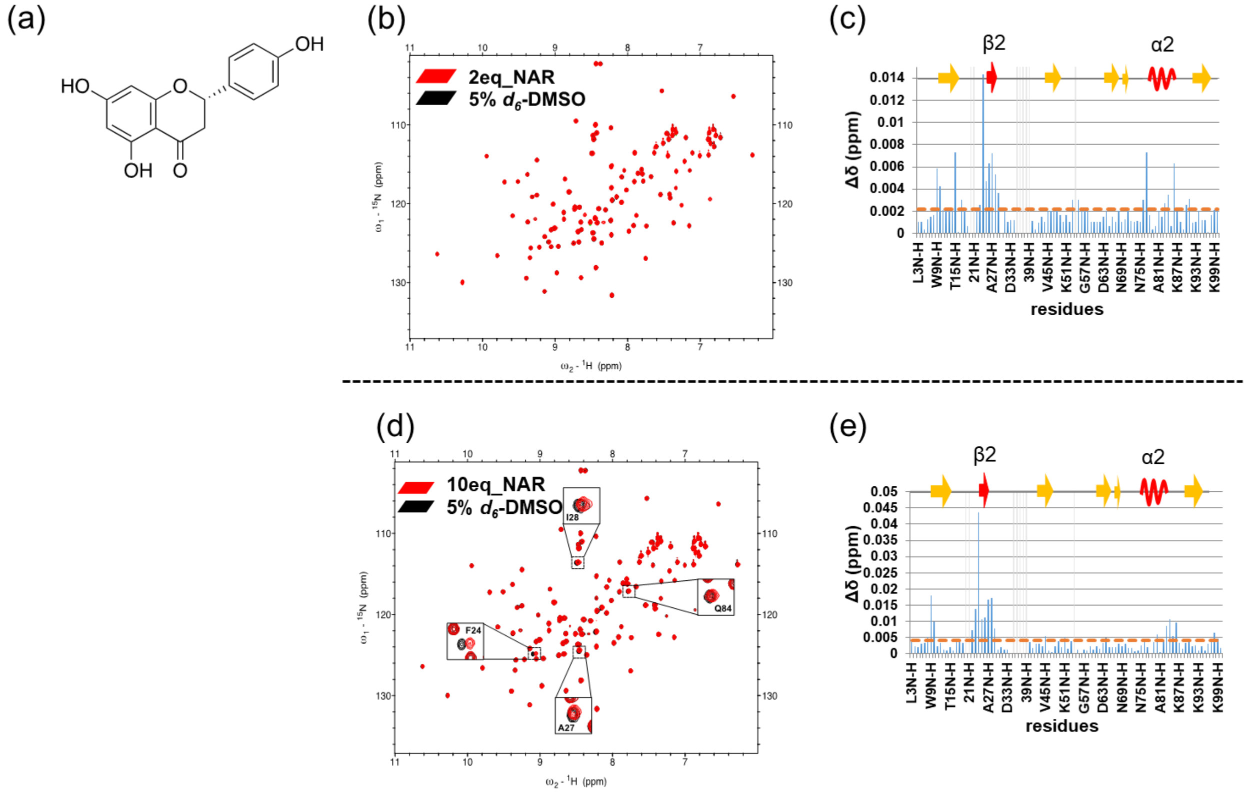

(B) ZO1-NRG

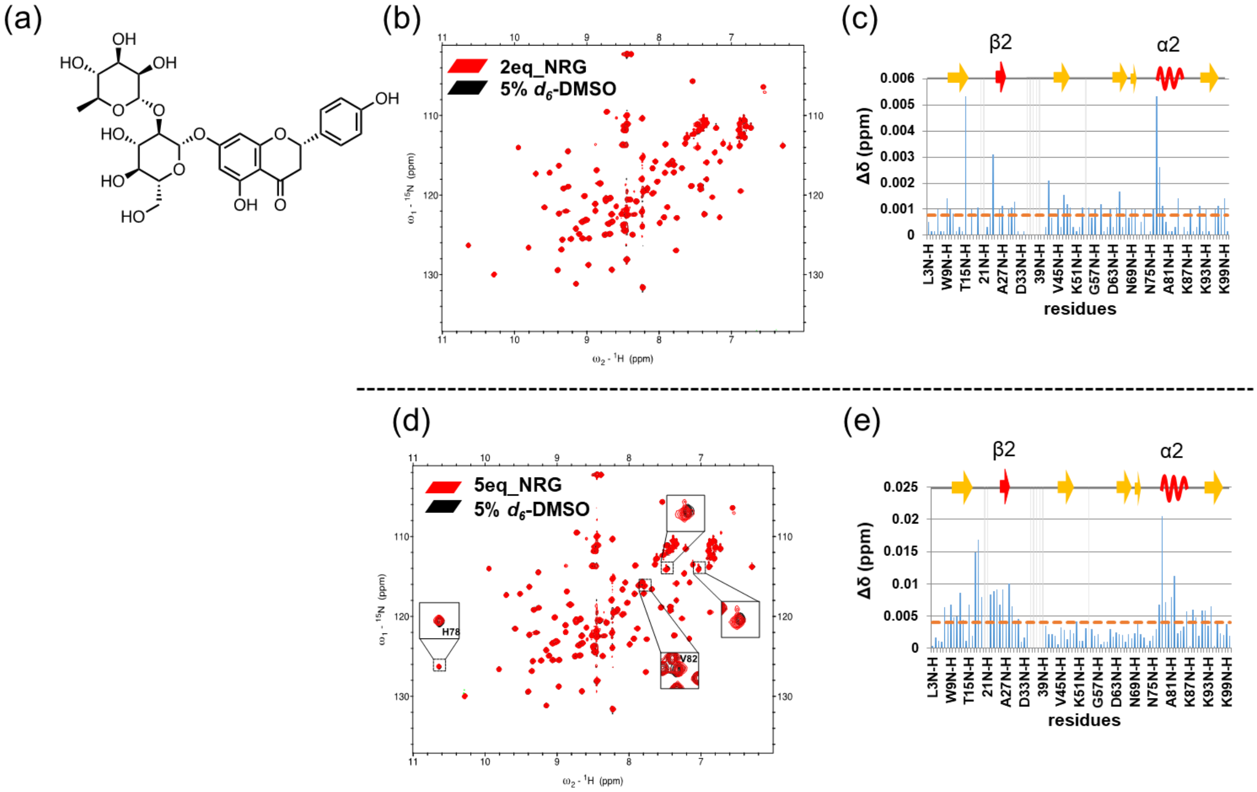

(C) ZO1-NRT

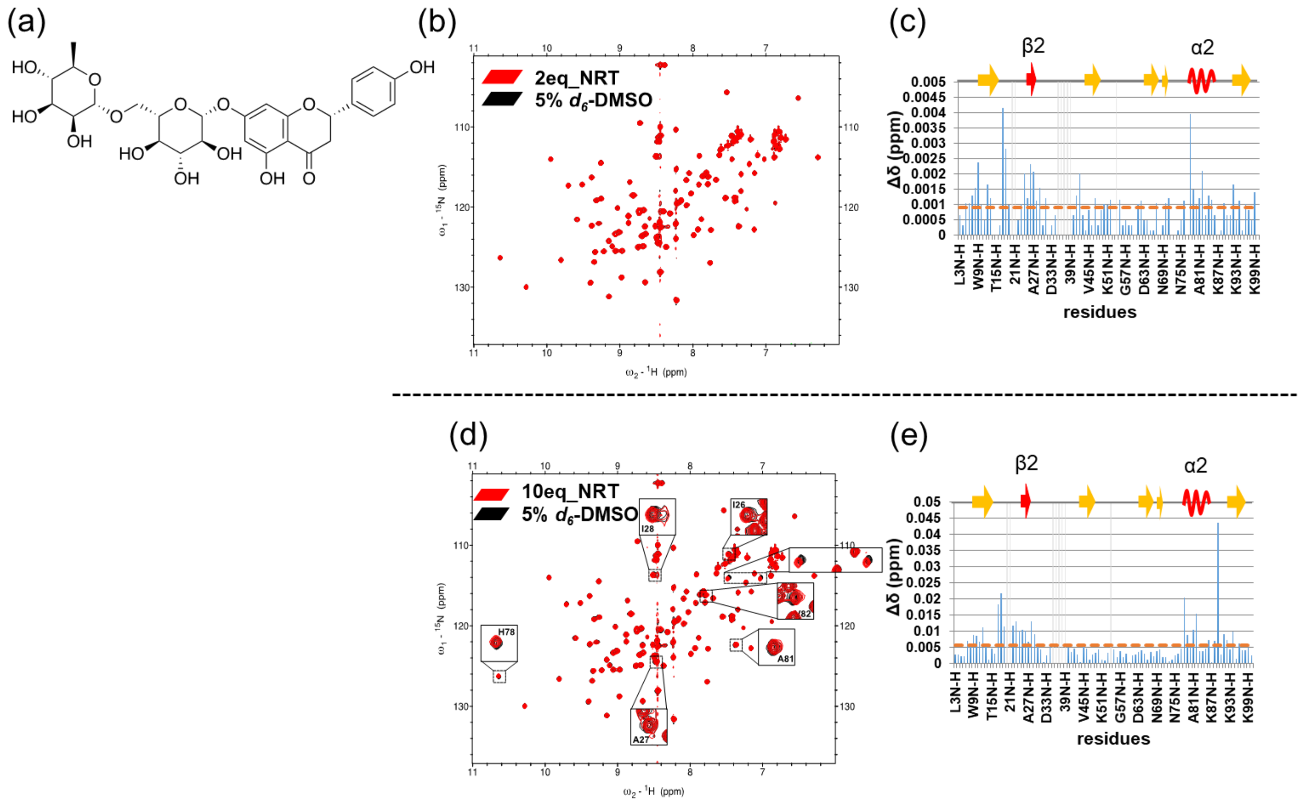

(D) ZO1-HST

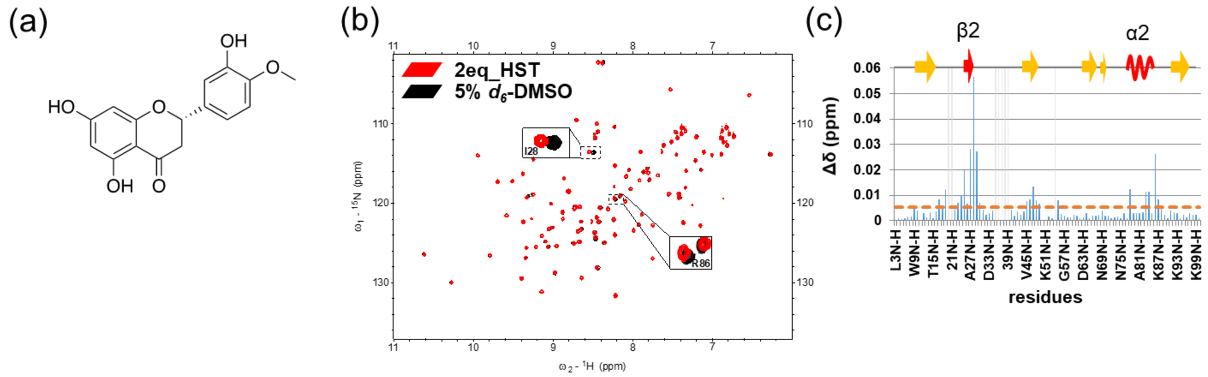

(E) ZO1-NHD

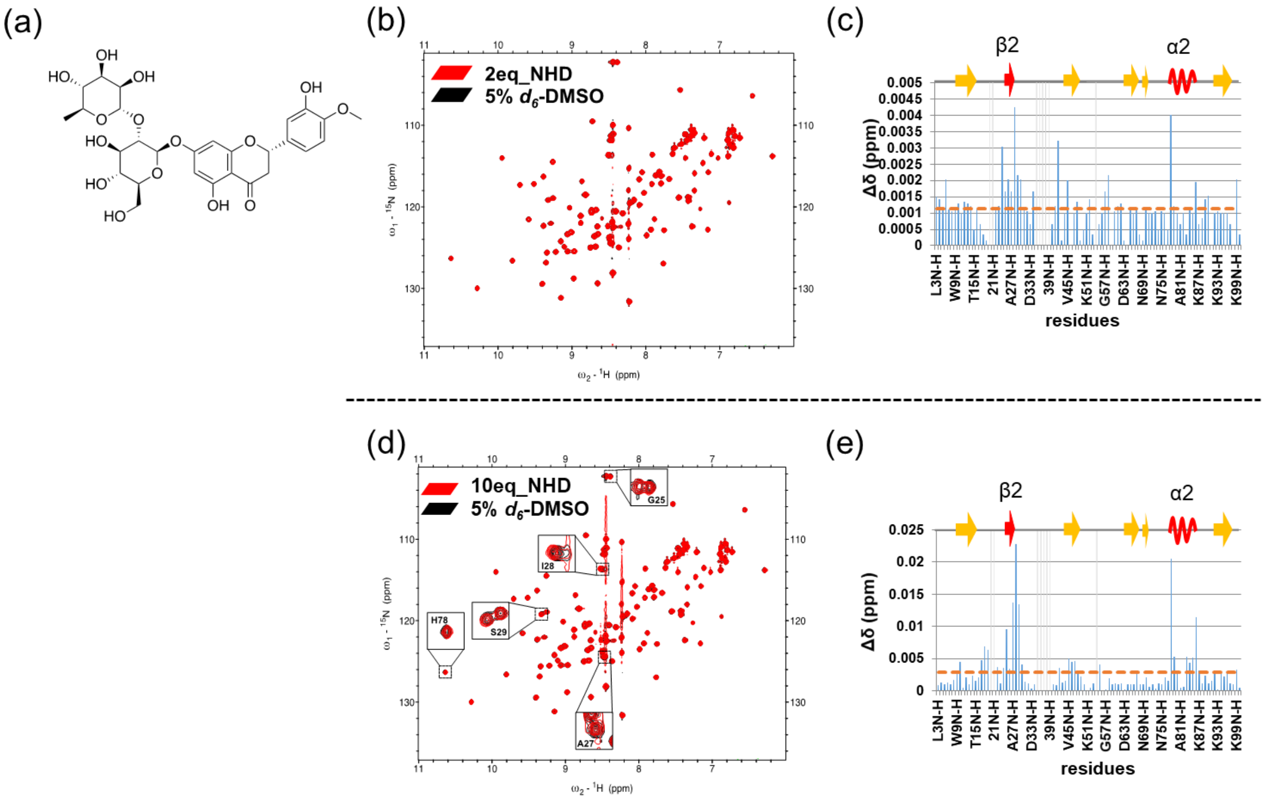

(F) ZO1-HSD

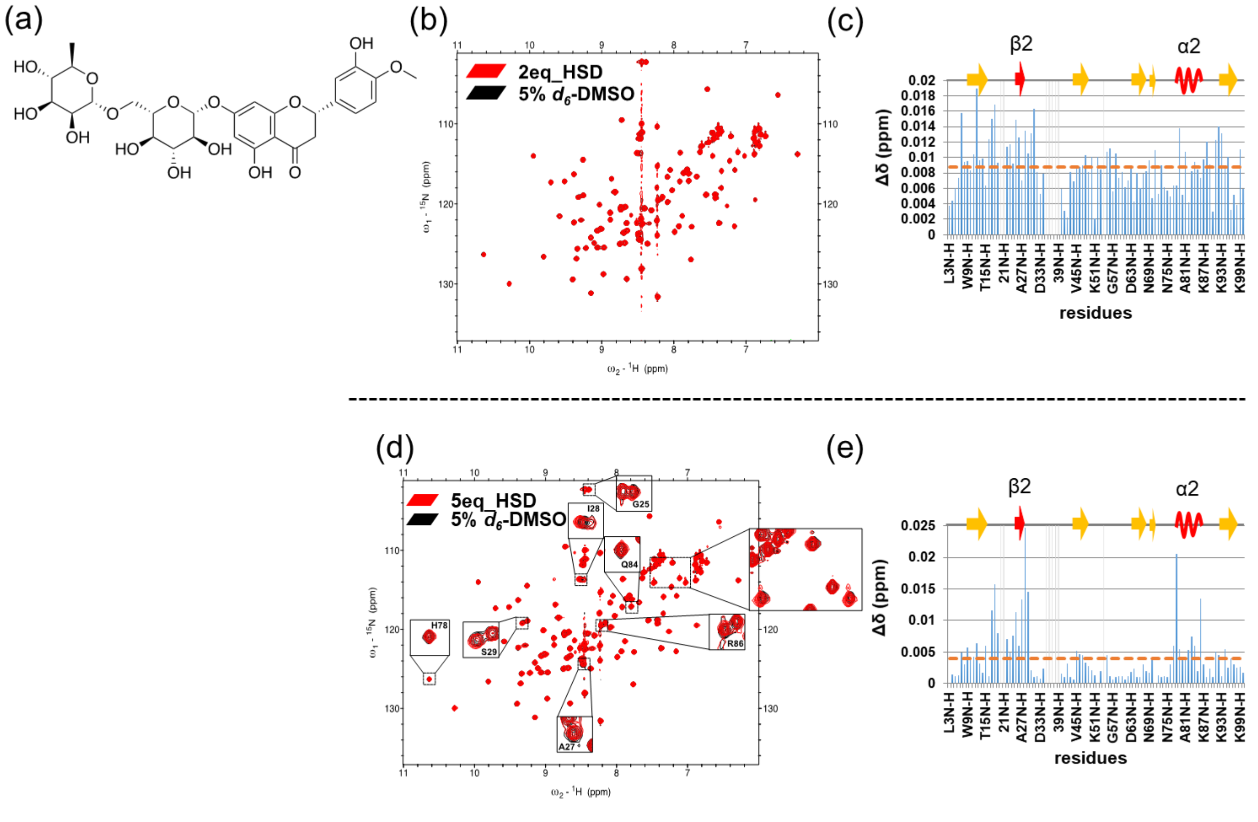

(G) ZO1-QUE

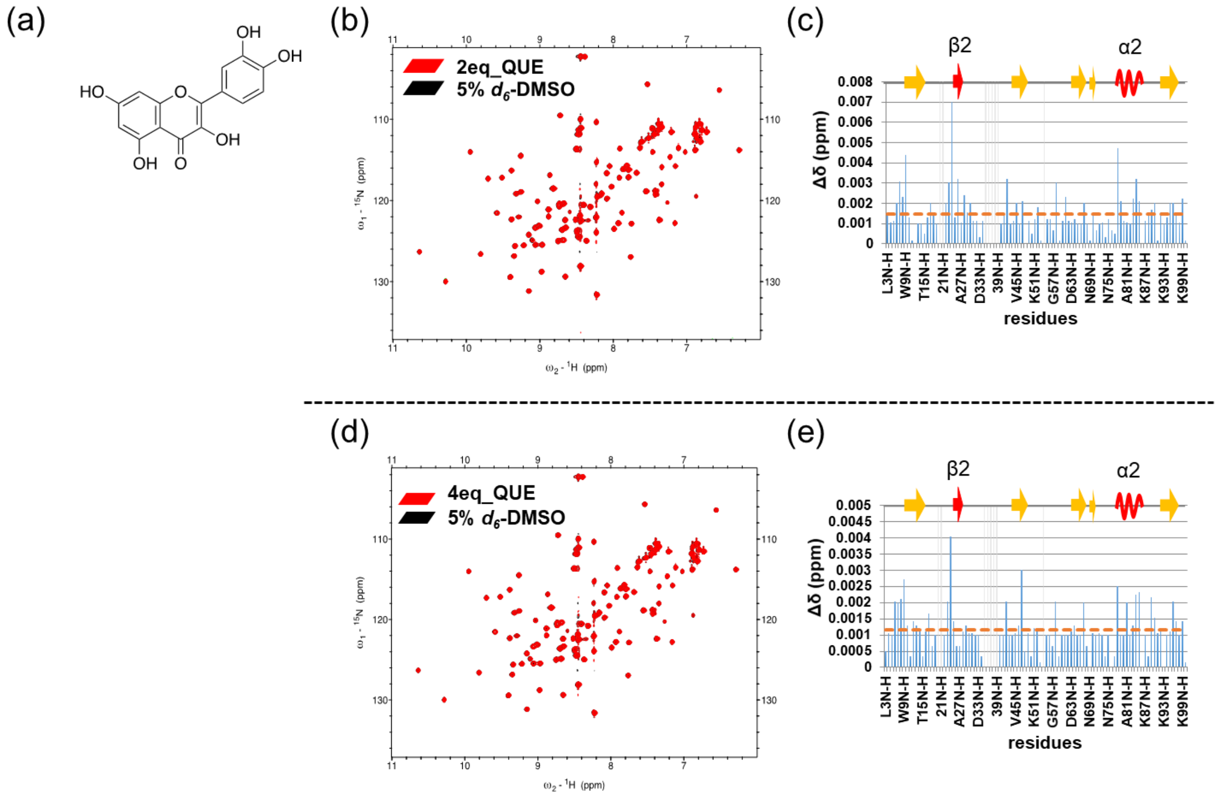

(H) ZO1-RUT

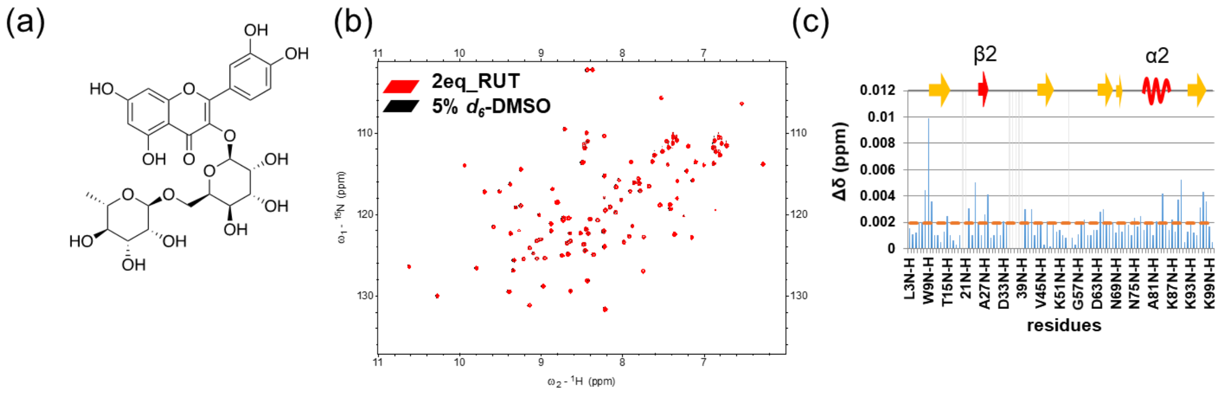

Supplementary Figure S7. Direct interaction between ZO-1(PDZ1) and the flavonoids. (a) Chemical structure of the flavonoids titrated. (b), (d) Overlaid HSQC spectra of 0.1 mM ZO-1(PDZ1) in the absence (black) and presence of 2 or 10 equivalent (red) of NAR (A), NRG (B) and NRT (C), 2 equivalent of HST (D), 2 or 10 equivalent of NHD (E), 2 or 5 equivalent of hesperidin (F), 2 or 4 equivalent of QUE (G) and 2 equivalent of RUT (H), respectively. (c), (e) Normalized chemical shift changes in the presence of 2 or 10 equivalent of NAR (A), NRG (B) and NRT (C), 2 equivalent of HST (D), 2 or 10 equivalent of NHD (E), 2 or 5 equivalent of HSD (F), 2 or 4 equivalent of QUE (G) and 2 equivalent of RUT (H).

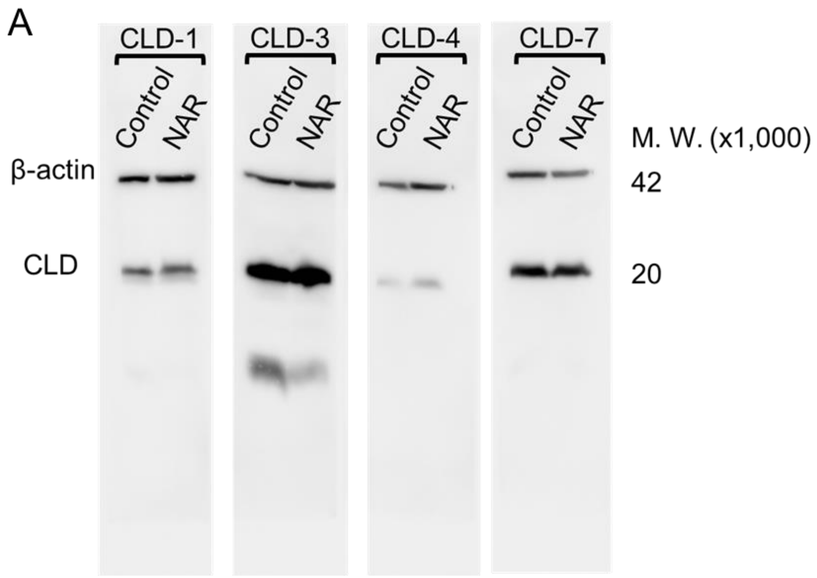

B

	1-Control	1-NAR	3-Control	3-NAR	4-Control	4-NAR	7-Control	7-NAR
β-actin	10298	12159	7206	10395	10787.6	12474	9696	6625
CLD-X	8619	11399	1587	2703.23	31611.7	34191.3	17677	15773
Relative level(%)	100	112	100	118	100	94	100	131

Supplementary Figure S8. Effects of NAR on CLD1, 3, 4, 7 expression in MDCK II cells.

Cells were treated with NAR at a concentration of 100 μM for 48 hours.

(A) Western blotting analysis of CLD-1, -3, -4 and -7 expression in cell lysates from control and 100 μM NAR-treated MDCK II cells. (B) Western blotting densitometry is above table.

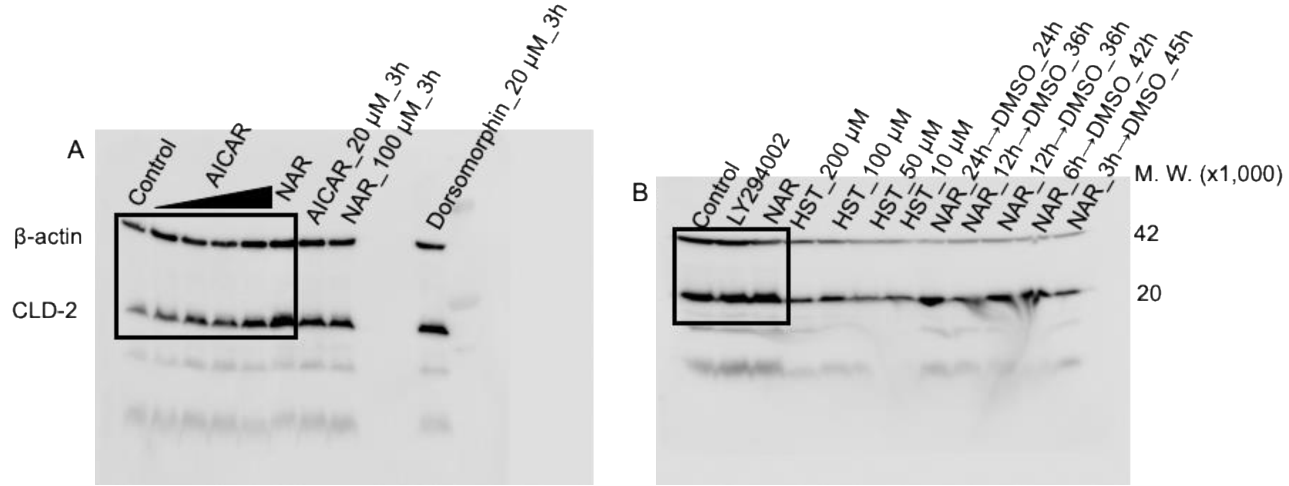

Supplementary Figure S9. Effects of compounds on CLD-2 expression in MDCK II cells.

Cells were treated with compounds at each concentration for 48 hours. (A), (B) Bands in the black line is shown in Fig. 7.

compound	LNX1-PDZ2	ZO1-PDZ1				effect on CLD2
	interaction	interaction	Δδave.(2eq) [ppm]	interaction	Δδave.(X eq) [ppm]	
Naringenin	±	±	0.0022	++	0.0043 (10eq)	+++
Naringin	±	±	0.0008	++	0.0041 (5eq)	++
Narirutin	±	±	0.0009	++	0.0057 (10eq)	±
Hesperetin	±	++	0.0052	no data	no data	- -
Hesperidin	±	±	0.0087	++	0.0040 (5eq)	++
Neohesperidin	±	±	0.0011	+	0.0029 (10eq)	±
Quercetin	precipitation	precipitation	0.0015	precipitation	0.0012 (4eq)	- - -
Rutin	no data	±	0.0019	no data	no data	±

Supplementary Table S1. Effects of flavonoids on NMR and the amount of CLD2.

We show interactions between ZO-1 / LNX-1 and flavonoids, and effects of CLD2 by flavonoids.

	research information	QUE concentration	time	cell type	effect on CLD	toxicity	other signaling
1	The Journal of Nutrition, Volume 138, Issue 6, 2008, 1067–1073.	200 μM	24 h	Caco-2	unaffect CLD1, 3, 7	-	not involved in ML-7
2	The Journal of Nutrition, Volume 139, Issue 5, 2009, 965–974.	100 μM	0-48 h	Caco-2	increase CLD4	no	further investigation
3	Nutrients 2015, 7(6), 4578-4592.	0.5-100 μM	24 h	A549	decrease CLD2	-	not involved in ERK, Akt
4	Am J Transl Res. 2019; 11(8): 4683–4695.	25 μmol/kg	twice/day, 3 days	rat brain tissue	increase CLD5	no	activate Wnt-β-catenin
5	Biological Trace Element Research, 2020	75 mg/kg	at 3-h intervals, 35 days	mouse testis	decrease CLD11	no	further investigation
6	this research	100 μM	48 h	MDCK II	decrease CLD2	yes	further investigation

Supplementary Table S2. Effects of quercetin (QUE) on some CLD.

Result of research is the above.
